# RBBP4 is an epigenetic barrier for the induced transition of pluripotent stem cells into totipotent 2C-like cells

**DOI:** 10.1093/nar/gkad219

**Published:** 2023-04-06

**Authors:** Wangfang Ping, Yingliang Sheng, Gongcheng Hu, Hongxin Zhong, Yaoyi Li, YanJiang Liu, Wei Luo, Chenghong Yan, Yulin Wen, Xinxiu Wang, Qing Li, Rong Guo, Jie Zhang, Ake Liu, Guangjin Pan, Hongjie Yao

**Affiliations:** State Key Laboratory of Respiratory Disease, The First Affiliated Hospital of Guangzhou Medical University, Guangzhou Laboratory, Guangzhou Medical University; Guangzhou Institutes of Biomedicine and Health, Chinese Academy of Sciences, Guangzhou, China; CAS Key Laboratory of Regenerative Biology, Guangdong Provincial Key Laboratory of Stem Cell and Regenerative Medicine, Guangzhou Institutes of Biomedicine and Health, Chinese Academy of Sciences, Guangzhou, China; University of Chinese Academy of Sciences, Beijing, China; Institute of Stem Cell and Regeneration, Chinese Academy of Sciences, Beijing, China; State Key Laboratory of Respiratory Disease, The First Affiliated Hospital of Guangzhou Medical University, Guangzhou Laboratory, Guangzhou Medical University; Guangzhou Institutes of Biomedicine and Health, Chinese Academy of Sciences, Guangzhou, China; CAS Key Laboratory of Regenerative Biology, Guangdong Provincial Key Laboratory of Stem Cell and Regenerative Medicine, Guangzhou Institutes of Biomedicine and Health, Chinese Academy of Sciences, Guangzhou, China; Institute of Stem Cell and Regeneration, Chinese Academy of Sciences, Beijing, China; Division of Life Sciences and Medicine, University of Science and Technology of China, Hefei, China; State Key Laboratory of Respiratory Disease, The First Affiliated Hospital of Guangzhou Medical University, Guangzhou Laboratory, Guangzhou Medical University; Guangzhou Institutes of Biomedicine and Health, Chinese Academy of Sciences, Guangzhou, China; State Key Laboratory of Respiratory Disease, The First Affiliated Hospital of Guangzhou Medical University, Guangzhou Laboratory, Guangzhou Medical University; Guangzhou Institutes of Biomedicine and Health, Chinese Academy of Sciences, Guangzhou, China; CAS Key Laboratory of Regenerative Biology, Guangdong Provincial Key Laboratory of Stem Cell and Regenerative Medicine, Guangzhou Institutes of Biomedicine and Health, Chinese Academy of Sciences, Guangzhou, China; University of Chinese Academy of Sciences, Beijing, China; Institute of Stem Cell and Regeneration, Chinese Academy of Sciences, Beijing, China; State Key Laboratory of Respiratory Disease, The First Affiliated Hospital of Guangzhou Medical University, Guangzhou Laboratory, Guangzhou Medical University; Guangzhou Institutes of Biomedicine and Health, Chinese Academy of Sciences, Guangzhou, China; CAS Key Laboratory of Regenerative Biology, Guangdong Provincial Key Laboratory of Stem Cell and Regenerative Medicine, Guangzhou Institutes of Biomedicine and Health, Chinese Academy of Sciences, Guangzhou, China; Institute of Stem Cell and Regeneration, Chinese Academy of Sciences, Beijing, China; State Key Laboratory of Respiratory Disease, The First Affiliated Hospital of Guangzhou Medical University, Guangzhou Laboratory, Guangzhou Medical University; Guangzhou Institutes of Biomedicine and Health, Chinese Academy of Sciences, Guangzhou, China; CAS Key Laboratory of Regenerative Biology, Guangdong Provincial Key Laboratory of Stem Cell and Regenerative Medicine, Guangzhou Institutes of Biomedicine and Health, Chinese Academy of Sciences, Guangzhou, China; University of Chinese Academy of Sciences, Beijing, China; Institute of Stem Cell and Regeneration, Chinese Academy of Sciences, Beijing, China; State Key Laboratory of Respiratory Disease, The First Affiliated Hospital of Guangzhou Medical University, Guangzhou Laboratory, Guangzhou Medical University; Guangzhou Institutes of Biomedicine and Health, Chinese Academy of Sciences, Guangzhou, China; CAS Key Laboratory of Regenerative Biology, Guangdong Provincial Key Laboratory of Stem Cell and Regenerative Medicine, Guangzhou Institutes of Biomedicine and Health, Chinese Academy of Sciences, Guangzhou, China; Institute of Stem Cell and Regeneration, Chinese Academy of Sciences, Beijing, China; State Key Laboratory of Respiratory Disease, The First Affiliated Hospital of Guangzhou Medical University, Guangzhou Laboratory, Guangzhou Medical University; Guangzhou Institutes of Biomedicine and Health, Chinese Academy of Sciences, Guangzhou, China; CAS Key Laboratory of Regenerative Biology, Guangdong Provincial Key Laboratory of Stem Cell and Regenerative Medicine, Guangzhou Institutes of Biomedicine and Health, Chinese Academy of Sciences, Guangzhou, China; Institute of Stem Cell and Regeneration, Chinese Academy of Sciences, Beijing, China; State Key Laboratory of Respiratory Disease, The First Affiliated Hospital of Guangzhou Medical University, Guangzhou Laboratory, Guangzhou Medical University; Guangzhou Institutes of Biomedicine and Health, Chinese Academy of Sciences, Guangzhou, China; CAS Key Laboratory of Regenerative Biology, Guangdong Provincial Key Laboratory of Stem Cell and Regenerative Medicine, Guangzhou Institutes of Biomedicine and Health, Chinese Academy of Sciences, Guangzhou, China; University of Chinese Academy of Sciences, Beijing, China; Institute of Stem Cell and Regeneration, Chinese Academy of Sciences, Beijing, China; State Key Laboratory of Respiratory Disease, The First Affiliated Hospital of Guangzhou Medical University, Guangzhou Laboratory, Guangzhou Medical University; Guangzhou Institutes of Biomedicine and Health, Chinese Academy of Sciences, Guangzhou, China; CAS Key Laboratory of Regenerative Biology, Guangdong Provincial Key Laboratory of Stem Cell and Regenerative Medicine, Guangzhou Institutes of Biomedicine and Health, Chinese Academy of Sciences, Guangzhou, China; University of Chinese Academy of Sciences, Beijing, China; Institute of Stem Cell and Regeneration, Chinese Academy of Sciences, Beijing, China; State Key Laboratory of Respiratory Disease, The First Affiliated Hospital of Guangzhou Medical University, Guangzhou Laboratory, Guangzhou Medical University; Guangzhou Institutes of Biomedicine and Health, Chinese Academy of Sciences, Guangzhou, China; CAS Key Laboratory of Regenerative Biology, Guangdong Provincial Key Laboratory of Stem Cell and Regenerative Medicine, Guangzhou Institutes of Biomedicine and Health, Chinese Academy of Sciences, Guangzhou, China; University of Chinese Academy of Sciences, Beijing, China; Institute of Stem Cell and Regeneration, Chinese Academy of Sciences, Beijing, China; State Key Laboratory of Respiratory Disease, The First Affiliated Hospital of Guangzhou Medical University, Guangzhou Laboratory, Guangzhou Medical University; Guangzhou Institutes of Biomedicine and Health, Chinese Academy of Sciences, Guangzhou, China; CAS Key Laboratory of Regenerative Biology, Guangdong Provincial Key Laboratory of Stem Cell and Regenerative Medicine, Guangzhou Institutes of Biomedicine and Health, Chinese Academy of Sciences, Guangzhou, China; Institute of Stem Cell and Regeneration, Chinese Academy of Sciences, Beijing, China; State Key Laboratory of Respiratory Disease, The First Affiliated Hospital of Guangzhou Medical University, Guangzhou Laboratory, Guangzhou Medical University; Guangzhou Institutes of Biomedicine and Health, Chinese Academy of Sciences, Guangzhou, China; CAS Key Laboratory of Regenerative Biology, Guangdong Provincial Key Laboratory of Stem Cell and Regenerative Medicine, Guangzhou Institutes of Biomedicine and Health, Chinese Academy of Sciences, Guangzhou, China; Institute of Stem Cell and Regeneration, Chinese Academy of Sciences, Beijing, China; Department of Life Sciences, Changzhi University, Changzhi, China; CAS Key Laboratory of Regenerative Biology, Guangdong Provincial Key Laboratory of Stem Cell and Regenerative Medicine, Guangzhou Institutes of Biomedicine and Health, Chinese Academy of Sciences, Guangzhou, China; University of Chinese Academy of Sciences, Beijing, China; Institute of Stem Cell and Regeneration, Chinese Academy of Sciences, Beijing, China; State Key Laboratory of Respiratory Disease, The First Affiliated Hospital of Guangzhou Medical University, Guangzhou Laboratory, Guangzhou Medical University; Guangzhou Institutes of Biomedicine and Health, Chinese Academy of Sciences, Guangzhou, China; CAS Key Laboratory of Regenerative Biology, Guangdong Provincial Key Laboratory of Stem Cell and Regenerative Medicine, Guangzhou Institutes of Biomedicine and Health, Chinese Academy of Sciences, Guangzhou, China; University of Chinese Academy of Sciences, Beijing, China; Institute of Stem Cell and Regeneration, Chinese Academy of Sciences, Beijing, China

## Abstract

Cellular totipotency is critical for whole-organism generation, yet how totipotency is established remains poorly illustrated. Abundant transposable elements (TEs) are activated in totipotent cells, which is critical for embryonic totipotency. Here, we show that the histone chaperone RBBP4, but not its homolog RBBP7, is indispensable for maintaining the identity of mouse embryonic stem cells (mESCs). Auxin-induced degradation of RBBP4, but not RBBP7, reprograms mESCs to the totipotent 2C-like cells. Also, loss of RBBP4 enhances transition from mESCs to trophoblast cells. Mechanistically, RBBP4 binds to the endogenous retroviruses (ERVs) and functions as an upstream regulator by recruiting G9a to deposit H3K9me2 on ERVL elements, and recruiting KAP1 to deposit H3K9me3 on ERV1/ERVK elements, respectively. Moreover, RBBP4 facilitates the maintenance of nucleosome occupancy at the ERVK and ERVL sites within heterochromatin regions through the chromatin remodeler CHD4. RBBP4 depletion leads to the loss of the heterochromatin marks and activation of TEs and 2C genes. Together, our findings illustrate that RBBP4 is required for heterochromatin assembly and is a critical barrier for inducing cell fate transition from pluripotency to totipotency.

## INTRODUCTION

In mice, zygotes and 2-cell (2C) blastomeres have the capacity to develop into all embryonic and extraembryonic cell types, exhibiting a totipotent state (1,2). The totipotency gradually decreases as development proceeds. The first lineage segregation occurs at the eight-cell stage, which separates the inner cell mass (ICM) from the trophectoderm (TrE) (3,4). Then second lineage segregation occurs at the blastocyst stage, when the ICM is divided into epiblast (EPI) and primitive endoderm (PE) (5). The EPI, PE and TrE are the precursors of the embryo proper, yolk sac and placenta, respectively (6,7). In contrast, lineage restriction has not been formed in human blastocysts (8–10). Human naïve pluripotent stem cells share properties with pre-implantation blastocysts and have been reported to have the potential to differentiate into both embryonic and extraembryonic trophoblast lineages (10–16). Pluripotent mESCs are isolated from the ICM (17,18) and contribute to all three germ layers of embryos but rarely to TrE-derived lineages (4,19). Overexpression of the transcription factor (TF) genes of the TrE (such as *Cdx2*, *Elf5* and *Eomes*) could lead to the direct conversion of mESCs into TrE-like cells (20–24), but lineage transition remains insufficient. In fact, the first cell lineage segregation during development is so refractory that even naïve mESCs cannot fully overcome it (25,26). Therefore, capturing totipotent cells is vital for giving rise to both ICM- and TrE-derived lineages.

A rare subset of cells that resembles 2C stage embryos (known as 2C-like cells, 2CLCs) has been identified among cultured mESCs, and several TF genes (such as *Dux*, *Zscan4* and *Nelfa*) have been found to facilitate the generation of 2CLCs from mESCs (27–31). Extended or expanded pluripotent stem cells (EPSCs) were reported to be another type of cells with both embryonic and extraembryonic developmental potential (32,33). However, a recent study has brought into question whether EPSCs have the ability to differentiate into the trophoblast lineages (4). Several laboratories have established various types of totipotent-like stem cells by combining different chemical molecules (34–37), yet the totipotency of these cells remains to be validated. Moreover, currently, the epigenetic barrier in regulating the transition from pluripotency to totipotency remains unclear.

Histone chaperones can modulate chromatin structure and function in a context-dependent manner (38). RBBP4 and RBBP7 are highly homologous H3/H4 histone chaperones (sharing 89% homology in amino acid sequence) in mice and humans (39,40). Moreover, RBBP4 and RBBP7 are components of various epigenetic complexes involved in histone modifications and chromatin remodeling such as polycomb repressive complex 2 (PRC2) (41), nucleosome remodeling and deacetylase (NuRD) complex (42) and nucleosome remodeling factor (NURF) complex (43). Nevertheless, whether RBBP4 and RBBP7 exert similar functions is still controversial (44–48). In addition, RBBP4 belongs to the chromatin assembly factor-1 (CAF-1) complex, which also contains the CHAF1A and CHAF1B subunits, and loss of *Chaf1a/Chaf1b* could promote totipotency (49). Therefore, we wondered whether RBBP4 and RBBP7 also participate in regulating cell fate transition from pluripotency to totipotency.

Here, we report that the histone chaperone RBBP4, but not RBBP7, is necessary for maintaining the identity of mESCs. Acute degradation of RBBP4 in mESCs activates the 2C transcriptional program including transposable elements (TEs) and 2C genes, and enhances trophoblast formation. Remarkably, RBBP4 is co-enriched at all three classes of ERVs (ERV1, ERVK and ERVL) with either H3K9me2 or H3K9me3. Intriguingly, RBBP4 could simultaneously recruit G9a and KAP1 to deposit H3K9me2 and H3K9me3 on the TEs, respectively. Moreover, RBBP4 increases nucleosome density at TE sites, and RBBP4 depletion reduces chromatin binding of both H3K9me2 and H3K9me3, and reprograms the pluripotent stem cells toward 2C-like totipotent cells.

## MATERIALS AND METHODS

### Cell culture

The 46C mESCs were cultured on 0.1% gelatin-coated plates with the mESCs medium containing high-glucose Dulbecco's modified Eagle's medium (DMEM, HyClone, SH30249.01), 15% fetal bovine serum (FBS; Gibco, 10082147), 1× GlutaMAX (Gibco, 35050061), 1× non-essential amino acids (Gibco, 11140050), 1× sodium pyruvate (Gibco, 11360070), 0.1 mM 2-mercaptoethanol (Gibco, 21985023), 3 μM Chir99021 (Selleck, S2924), 1 μM PD0325901 (Selleck, S1036) and 1000 U/ml leukemia inhibitory factor (LIF). HEK293T cells (ATCC, CRL-1126) were maintained in high-glucose DMEM supplemented with 10% FBS (Natocor, SFBE). All cell lines were cultured under 5% CO_2_ at 37°C. RBBP4 or RBBP7 degradation was induced by treating mESCs with a final concentration of 500 μM indole-3-acetic acid (IAA; Sigma, I5148).

### Generation of *Rbbp4*-auxin-induced degron (*Rbbp4*-AID) or *Rbbp7*-AID stable mESCs

We used the clustered regularly interspaced short palindromic repeats (CRISPR)/CRISPR-associated 9 (Cas9) system to construct the gene-edited cell lines. To construct the *Rbbp4* or *Rbbp7* donor plasmid, a DNA fragment containing the stop site (∼1 kb), which was amplified by polymerase chain reaction (PCR) from the genomic DNA of 46C mESCs, was cloned into a pEASY-Blunt plasmid (TransGen Biotech, CB101). After creating a restriction enzyme BamHI site by inverse PCR, a DNA fragment containing the AID–mCherry tag linked to a SV40 promoter-driven neomycin/kanamycin resistance gene (AID–mCherry-NeoR) was cloned into the donor plasmid. The AID–mCherry-NeoR backbone was cut from the PTJ58 vector by BamHI. The specific guide RNA (gRNA) targeting the 3′-end sequences of the mouse *Rbbp4* or *Rbbp7* gene were designed and inserted into a pX330 plasmid (Addgene, 42230), respectively. The single guide RNA (sgRNA) oligos are available in Supplementary Table S1.

The parental mESCs expressing an *OsTIR1* were established by co-transfection of the plasmids pEN396 (Addgene, 92142) and pX330-EN1201 (Addgene, 92144) expressing a gRNA targeting the *Tigre* locus using FuGENE6 Transfection Reagent (Promega, E2691) following the manufacturer's instructions. Clones were selected with 1 μg/ml puromycin for 3 days. After validation of the *OsTIR1* expression cassette integration into the *Tiger* locus, the donor plasmids and their corresponding sgRNA targeting *Rbbp4* or *Rbbp7* were co-transfected into the parental cell lines. The transfected cells were selected with 200 μg/ml G418 for 5 days. Colonies were manually picked up and were further validated by PCR for homozygous insertion of the sequences encoding AID–mCherry.

### Plasmid construction and lentivirus production

For overexpression, *Rbbp4* and *Rbbp7* cDNAs were cloned into the pSin-Flag-Avitag vector with a puromycin resistance gene. For *Rbbp4* knockdown, the shRNA oligos targeting *Rbbp4* were inserted into the lentiviral vector pLKO.1. All sequences of the plasmids were validated by Sanger sequencing before using for further experiments. For lentivirus generation, HEK293T cells were plated, cultured overnight and then co-transfected with pSin or pLKO.1 vector containing target genes together with the packaging plasmids psPAX2 and pMD2.G by using polyethyleneimine (PEI; Polysciences, 24765–2). The culture medium was changed after 12 h. The viral supernatants were collected 48 h post-transfection. After filtration through a 0.45 μm filter, the supernatants were used to infect mESCs. The sequences of shRNA oligos used in this study are listed in Supplementary Table S2.

### Generation of *in vivo* biotinylated mESCs

The 46C mESCs were infected with lenti-birAV5 lentivirus and selected with 5 μg/ml blasticidin for 4 days. Then BirAV5-overexpressing cells were infected with pSin-FLAG-Avitag, pSin-Flag-Avitag-*Rbbp4* and pSin-Flag-Avitag-*Rbbp7* lentivirus, respectively. Thereafter, the cells were selected with 2 μg/ml puromycin for 3 days. Finally, *in vivo* biotinylation of RBBP4 and RBBP7 was detected with anti-Biotin antibody (Cell Signaling Technology, 7075).

### Trophoblast derivation from mESCs

Cell culture plates were pre-coated with 0.3% gelatin. *Cdx2*-tdTomato mESCs were trypsinized and then resuspended in trophoblast stem cell (TSC) medium containing RPMI1640 (Gibco, 11875093), 20% FBS, 1× GlutaMAX, 1× sodium pyruvate, 1× penicillin–streptomycin, 0.1 mM 2-mercaptoethanol, 2 ng/ml transforming growth factor-β1 (TGFβ1; Sino Biological, 10804-HNAC), 25 ng/ml fibroblast growth factor 4 (FGF4; Stem Cell, 78103) and 1 μg/ml heparin (Stem Cell, 07980). Then cells were replated at a density of 1000–5000 cells per well in 12-well plates and cultured in TSC medium. The culture medium was changed daily.

### Western blots

The cells were lysed in RIPA lysis buffer [150 mM NaCl, 1% Triton X-100, 1 mM EDTA, 0.1% sodium dodecylsulfate (SDS), 50 mM Tris–HCl (pH 7.4), 1% sodium deoxycholate, 1 mM phenylmethylsulfonyl fluoride (PMSF), 1× protease inhibitor cocktail and 1 mM dithiothreitol (DTT)] on ice for 30 min and the supernatants were obtained by centrifuging at 12000 rpm at 4°C for 10 min. The boiled proteins were separated by SDS–polyacrylamide gel electrophoresis (SDS–PAGE) and transferred onto polyvinyldifluoridene (PVDF) membranes. The membranes were incubated with primary antibodies at room temperature (RT) for 2 h or at 4°C overnight after blocking with 5% milk. The membranes were washed with phosphate-buffered saline (PBS) containing 0.1% Tween-20 and then incubated with secondary antibodies conjugated to horseradish peroxidase (HRP) at RT for 1 h. ECL solution was added and the signals were detected using a MiniChemi™ Chemiluminescence Imaging System (Beijing Sage Creation). The primary antibodies used herein are as follows: RBBP4 (Abcam, ab79416), RBBP7 (Abcam, ab259957), KAP1 (Abcam, ab22553), CHD4 (Abcam, ab70469), G9a (Cell Signaling Technology, 3306), HP1α (Cell Signaling Technology, 2616), β-Actin (Sigma, A2228) and glyceraldehyde phosphate dehydrogenase (GAPDH; KangChen Bio-tech, KC-5G5).

### Cell viability assay

Approximately 5000 cells were plated per well of 96-well plates. The next day, the cells were cultured in 100 μl of mES culture medium with or without IAA (this time point was recorded as day 0). The cell viability was detected at the indicated time points by using Cell Counting Kit-8 (CCK8, Beyotime, C0037) according to the manufacturer's instructions. In brief, 10 μl of CCK8 solution was added to each well and incubated for 1.5 h, and the absorbance at 450 nm was measured using a spectrophotometer.

### Apoptosis assay

Apoptosis assays were performed using the Annexin V-FITC Apoptosis Detection Kit (Beyotime, C1062) according to the manufacturer's instructions. In brief, 30000 cells were plated per well of 6-well plates and treated with 500 μM IAA or PBS for 24 h before collection. The supernatants of the culture medium were collected. The adherent cells were washed once with PBS, digested with trypsin without EDTA and collected together with the supernatants. The cells were centrifuged and washed once with PBS. The cells were resuspended in 100 μl of 1× Annexin V binding buffer. Then 5 μl of Annexin V–fluorescein isothiocyanate (FITC) was added to the cell suspension and incubated in the dark at RT for 10 min. Subsequently, 400 μl of 1× Annexin V binding buffer was added to each sample and the fluorescence intensity was detected using an LSRFortessa flow cytometer (BD Biosciences) within 1 h.

### Cell cycle analysis

A total of 40000 cells were plated per well of 6-well plates and treated with IAA for 24 h before collection. The cells were washed and then fixed with pre-cooled 75% ethanol overnight at 4°C. The cells were washed once with PBS, resuspended with 100 μl of PBS containing 0.25 μg of 4′,6-diamidino-2-phenylindole (DAPI; Beyotime, C1002) and incubated in the dark at RT for 15 min. Finally, 500 μl of PBS was added to each sample after washing and the fluorescence intensity was detected using an LSRFortessa flow cytometer (BD Biosciences).

### Alkaline phosphatase (AP) staining

AP staining was performed following the protocol of the BCIP/NBT Alkaline Phosphatase Color Development Kit (Beyotime, C3206). In brief, the cells were fixed with 4% paraformaldehyde (PFA) solution for 5 min and gently washed, followed by staining with BCIP/NBT solution in the dark at RT for 30 min.

### Biotin immunoprecipitation

Flag-Avitag- or Flag-Avitag-*Rbbp4*-overexpressing 46C mESCs were collected and lysed with cell lysis buffer [50 mM Tris–HCl (pH 7.9), 150 mM NaCl, 1% NP-40, 1 mM PMSF, 1× protease inhibitors, 1 mM DTT]. The supernatants were obtained by centrifugation at 12000 rpm at 4°C for 10 min and 1 mg of proteins were used for immunoprecipitation. A 15 μl aliquot of M280 Streptavidin Dynabeads (Invitrogen, 11205D) was added to the samples and incubated overnight at 4°C with gentle rotation. Beads were washed four times using 1 ml of cell lysis buffer at 4°C for 10 min each time. Immunoprecipitated proteins were visualized by western blots.

### RNA extraction and RT–qPCR analysis

To extract the total RNAs, the cells were lysed with TRIzol reagent (MRC, TR118), followed by addition of 1/5 volume of chloroform. After centrifugation at 12000 rpm at 4°C for 10 min, the total RNAs in the supernatants were precipitated with isopropanol. cDNAs were synthesized with HiScript^®^ III RT SuperMix for quantitative PCR (Vazyme, R323). Quantitative reverse transciption–PCR (RT–qPCR) was performed using SYBR Green mix (Genstar, A301) on the CFX96 system (Bio-Rad). Gene expression levels were normalized to *Gapdh*. The primers used in the RT–qPCR assays are listed in Supplementary Table S3.

### Immunofluorescent staining

Cells were fixed with 4% PFA at RT for 10 min, permeabilized with 0.5% Triton X-100 at RT for 10 min and blocked with 3% bovine serum albumin (BSA) at RT for 1 h. Samples were then incubated with primary antibody overnight at 4°C and subsequently with secondary antibody. A final concentration of 1 μg/ml DAPI (Beyotime, C1002) was used to stain nuclei. Fluorescence images were captured with a ZEISS Axio Observer 7. The EOMES antibody (Abcam, ab183991) used in this study was diluted 1:500.

### Bulk RNA-seq

The total RNAs were extracted as described above. RNA sequencing libraries were constructed by using a VAHTS mRNA-seq V3 Library Prep Kit (Vazyme, NR611) according to the manufacturer's instructions. In brief, mRNA capture beads were used to capture poly(A)-enriched RNAs from 1.5 μg of total RNAs and then the RNAs were fragmented at 85°C for 6 min. Next, the first-strand and second-strand cDNAs were synthesized. The cDNAs were purified with AMPure XP beads (Beckman Coulter, A63881), followed by end repair, adaptor-ligation, size selection and library amplification. Finally, the libraries were purified using AMPure XP beads and then sequenced on a NovaSeq 6000 sequencer.

### Single-cell RNA sequencing (scRNA-seq)

scRNA-seq libraries were prepared by using a DNBelab C Series Single-Cell Library Prep set (MGI, 940–000047-00). In brief, the dissociated cell suspensions were converted to barcoded scRNA-seq libraries through droplet encapsulation, emulsion breakage, mRNA captured bead collection, reverse transcription, cDNA amplification and purification. The libraries were sequenced on a DNBSEQ-T7 sequencer.

### Cleavage under targets and tagmentation (CUT&Tag)

CUT&Tag assays were performed as described previously with some modifications (50). Briefly, 1 × 10^5^ cells were washed twice with wash buffer [20 mM HEPES (pH 7.5), 150 mM NaCl, 0.5 mM spermidine and 1× protease inhibitors] by gentle pipetting. A 10 μl aliquot of concanavalin A-coated magnetic beads (Bangs Laboratories, BP531) was activated and added per sample and incubated at RT for 10 min. The supernatants were removed and bead-bound cells were resuspended in 100 μl of wash buffer containing 0.01% digitonin and 2 mM EDTA. The H3K9me2 antibody (Cell Signaling Technology, 4658) was added and incubated overnight at 4°C on a rotator. The beads were washed and resuspended in 300-wash buffer containing 300 mM NaCl with pG-Tn5 (Vazyme, S602) at RT for 1 h. The beads were washed and tagmentation was performed in 300-wash buffer supplemented with 10 mM MgCl_2_ at 37°C for 1 h. To stop the tagmentation reaction, 2.25 μl of 0.5 M EDTA, 2.75 μl of 10% SDS and 5 μl of proteinase K (20 mg/ml) were added and further incubated at 55°C for 2 h. Then the genomic DNAs were extracted by phenol–chloroform and subjected to PCR amplification using NEBNext High-Fidelity 2× PCR Master Mix (NEB, M0541S) for 13 cycles. The libraries were size-selected with 1.2× AMpure XP beads (Beckman Coulter, A63881) and sequenced on a NovaSeq 6000 sequencer.

### Cleavage under targets and release using nuclease (CUT&RUN)

CUT&RUN assays were performed as described previously with some modifications (51). Briefly, 1 × 10^5^ cells were harvested, washed and bound to activated concanavalin A-coated magnetic beads as for CUT&Tag. The bead-bound cells were incubated with primary antibody in 100 μl containing 0.01% digitonin wash buffer [20 mM HEPES (pH 7.5), 150 mM NaCl, 0.5 mM spermidine and 1× protease inhibitors] on a rotator at 4°C overnight. The primary antibodies used herein are as follows: H3K9me3 (Abcam, ab8898), KAP1 (Abcam, ab109545), CHD4 (Abcam, ab70469) and G9a (Cell Signaling Technology, 3306). After two washes, the beads were resuspended in 100 μl of wash buffer containing pAG-MNase and incubated at 4°C for 1 h. After two washes in wash buffer and one wash in low-salt rinse buffer [20 mM HEPES (pH 7.5), 0.5 mM spermidine and 1× protease inhibitors], the tubes were chilled to 0°C and the beads were resuspended in 100 μl of ice-cold calcium incubation buffer [20 mM HEPES (pH 7.5), 2 mM CaCl_2_]. The tubes were kept on ice 30 min, followed by immediate addition of STOP buffer [170 mM NaCl, 20 mM EDTA]. The beads were incubated at 37°C for 30 min and the liquid was removed to a fresh tube on a magnet stand. Then SDS and proteinase K were added and incubated at 55°C for 2 h. The genomic DNAs were extracted by phenol–chloroform and the sequencing libraries were prepared through VAHTS Universal DNA Library Prep Kit (Vazyme, ND607), and sequenced on a NovaSeq 6000 sequencer.

### Micrococcal nuclease sequencing (MNase-seq)

MNase-seq was performed as described previously with some modifications (52,53). In brief, 1 × 10^6^ cells were harvested, washed in ice-cold PBS and resuspended in 1 ml of ice-cold lysis buffer [10 mM Tris–HCl (pH 7.4), 10 mM NaCl, 3 mM MgCl_2_, 0.5% IGEPAL CA-630, 1× complete protease inhibitor cocktail] and rotated at 4°C for 15 min. Nuclei were then pelleted at 300 *g* at 4°C for 10 min and washed in 1 ml of digestion buffer [10 mM Tris–HCl (pH 7.4), 15 mM NaCl, 60 mM KCl]. MNase digestion was carried out in 100 μl of digestion buffer containing 2 mM CaCl_2_ and 2500 gel units of MNase (NEB, M0247S) with shaking at 37°C for 5 min. The reaction was stopped using an equal volume of stop buffer (digestion buffer containing 20 mM EDTA, 2 mM EGTA) before RNase A and proteinase K treatment. The gDNAs were extracted by phenol–chloroform. Purified DNAs were loaded on a 1.5% agarose gel to determine digestion efficiency. The mononucleosome–DNAs were purified by AMPure XP beads (Beckman Coulter, A63881). The sequencing libraries were prepared from 10 ng of purified mononucleosome–DNAs using the VAHTS^®^ Universal DNA Library Prep Kit (Vazyme, ND607) with 10 cycles of PCR, and sequenced on a NovaSeq 6000 sequencer.

### Chromatin immunoprecipitation sequencing (ChIP-seq)

Biotin ChIP-seq was performed as described (54). In brief, mESCs stably expressed Biotin–RBBP4 or Biotin–RBBP7 were cross-linked with 1% formaldehyde. The cross-linked cells were resuspended in SDS lysis buffer [1% SDS, 50 mM Tris–HCl (pH 8.0), 10 mM EDTA], sonicated and diluted 10-fold with ChIP dilution buffer [0.01% SDS, 1.1% Triton X-100, 1.2 mM EDTA, 16.7 mM Tris–HCl (pH 8.0), 167 mM NaCl], and then incubated with M280 Streptavidin Dynabeads (Invitrogen, 11205D) at 4°C overnight. Streptavidin Dynabeads-bound DNAs were subsequently washed once with wash buffer 1 (2% SDS), once with wash buffer 2 [50 mM HEPES (pH 7.5), 1 mM EDTA, 500 mM NaCl, 0.1% sodium deoxycholate, 1% Triton X-100], once with wash buffer 3 [10 mM Tris–HCl (pH 8.0), 1 mM EDTA, 250 mM LiCl, 0.5% NP-40, 0.5% sodium deoxycholate] and then twice with TE wash buffer [10 mM Tris–HCl (pH 8.0), 1 mM EDTA]. ChIPed DNAs were reverse-cross-linked and purified. The sequencing libraries were prepared through the VAHTS Universal DNA Library Prep Kit (Vazyme, ND607) and sequenced on a NovaSeq 6000 sequencer.

### Bulk RNA-seq analysis

To analyze the expression of genes and TEs, RNA-seq raw reads were trimmed using Trim_galore (v0.6.7) and aligned to the mouse genome (mm10 and Gencode gene annotation vM25) using STAR (v2.7.0d) (55) and RSEM (v1.3.3) (56); the counts for each gene or TE were calculated using TEtranscripts (57). The DESeq2 package (58) was used for differential expression analysis. Differentially expressed genes and TEs were defined by a Benjamini–Hochberg-corrected *P* <0.01 and an absolute fold change (FC) >2 (for genes) or an absolute FC >1.5 (for TEs).

For principal component analysis (PCA) (Supplementary Figure S6C), the alignment and quantification processes were conducted as described above. Batch effects were removed using ComBat with the sva package (59). Gene set enrichment analysis was performed using GSEA software (v4.1.0) (60).

### Data analysis of ChIP-seq, CUT&RUN and CUT&Tag

Raw reads were trimmed using Trim_galore (v0.6.7) and mapped to the mouse genome (mm10) using Bowtie2 (v2.2.5) (61). For TE analysis, the multimapped reads were retained, but only the best alignment of these reads was reported; in the case of multiple equivalent best alignments, only one random alignment is reported. Low mapping quality reads were filtered using SAMtools (62) with the options ‘-F 1804 -f 2 -q 30’, and potential PCR duplicates were removed by Sambamba (v0.7.1) (63). SAMtools was used to merge BAM files of biological replicates. Bigwig files were generated by DeepTools (v3.5.1) (64) with the parameter ‘-normalizeUsing RPGC -binSize 1’ and visualized in the Integrated Genomics Viewer (IGV) browser (65).

RBBP4, RBBP7, G9a and KAP1 peaks were called by MACS2 (v2.2.7.1) (66) with the parameters ‘-nomodel -p 0.01’. For peak calling of broad histone modifications, MACS2 was applied with the parameters ‘-broad -q 0.01’ for H3K9me2 and with the parameters ‘-nolambda -broad -q 0.01’ for H3K9me3. RBBP4 peaks were filtered for fold enrichment >2. Peaks were annotated using Homer (67). The Jaccard statistic representing the ratio of the intersection of two sets to the union of the two sets was calculated using bedtools (v2.30.0) (68). Differential peaks between distinct treatments were identified by the Diffbind pipeline and peaks with high confidence were chosen according to the thresholds of a *P* <0.05 and an absolute FC >1.5.

For analysis of TEs, repeat element annotations (RepeatMasker) were downloaded from the UCSC genome browser database (mm10). TEs shorter than 300 bp or those TEs with <50 copies were removed from the analysis. RBBP4 enrichment on TEs was calculated by dividing the observed value by the input and was expressed as the log_2_(fold enrichment). Heatmaps and pile ups were generated using DeepTools (v3.5.1).

### MNase-seq analysis

Raw reads were trimmed by Trim_galore (v0.6.7) and mapped to the mouse genome (mm10) using Bowtie2. DANPOS3 (69) was used to count the length distributions of fragments and to analyze nucleosome position and occupancy with the default parameters. For visualization, the output files were used to create a profile with R. In addition, the meta profiles and boxplot (Figure 5D) (e.g. around RBBP4-binding sites) were normalized for library size and other technical differences by dividing each profile by its median and then multiplying it by the median over all profile medians in the same plot (70).

Nucleosome-free regions were evaluated as previously described with some modifications (71). Regions spanning 1 kb upstream and downstream of the center of the curated RBBP4 peaks were divided into 25 bp bins. The curated genomic locations were ordered by ascending rank according to the tag counts from the central 200 bp. Sites were considered to be nucleosome depleted if the tag counts of their central 200 bp were smaller than that of the average 200 bp.

### Single-cell RNA-seq analysis

Smart-Seq2 read files were mapped to the mouse genome (mm10 and Gencode gene annotation vM25) using STAR (v2.7.0d) (55) and RSEM (v1.3.3) (56). Cells with >500 expressed genes and a Pearson's correlation >60% between cells were included for the following analysis.

The analysis of DNBeLab C4 scRNA-seq data followed: https://github.com/MGI-tech-bioinformatics/DNBelab_C_Series_HT_scRNA-analysis-software. Cells with <200 expressed genes detected or >10% of mitochondrial reads were filtered out.

Analysis of the filtered data was conducted in R (v4.2.2) using the Seurat suite (v4.1.1) (72). The integration of our DNBelab C4 data with published Smart-seq2 datasets was performed using Seurat's canonical correlation analysis (CCA) integration tool. For integration, the 2000 most variable genes were identified by ‘FindVariableFeatures’ with the following parameters: selection.method = ‘vst’, nfeatures = 2000. Integration anchors were identified based on these genes using the CCA integration tool with 30 dimensions as implemented in the ‘FindIntegrationAnchors’ function. The data were then integrated using ‘IntegrateData’ and scaled again using ‘SCT’. PCA with 40 principal components was performed by ‘RunPCA’, and uniform manifold approximation and projection (UMAP) coordinates were computed using the first 26 principal components. Merged datasets were clustered using Seurat's shared nearest-neighbor algorithm implemented with the ‘FindClusters’ function.

### Quantification and statistical analysis

Data are presented as mean values ± standard deviaition (SD) unless otherwise indicated in the figure legends. Sample numbers and experimental repeats are indicated in the figure legends. Statistical significance was determined by Student's *t*-test analysis (two-tailed) for two groups, unless otherwise indicated. Differences in means were considered statistically significant at *P* <0.05. Significance levels were: *P <0.05; **P <0.01; and ***P <0.001.

## RESULTS

### RBBP4, but not RBBP7, is indispensable for maintaining the identity of mESCs

**Figure 1.**
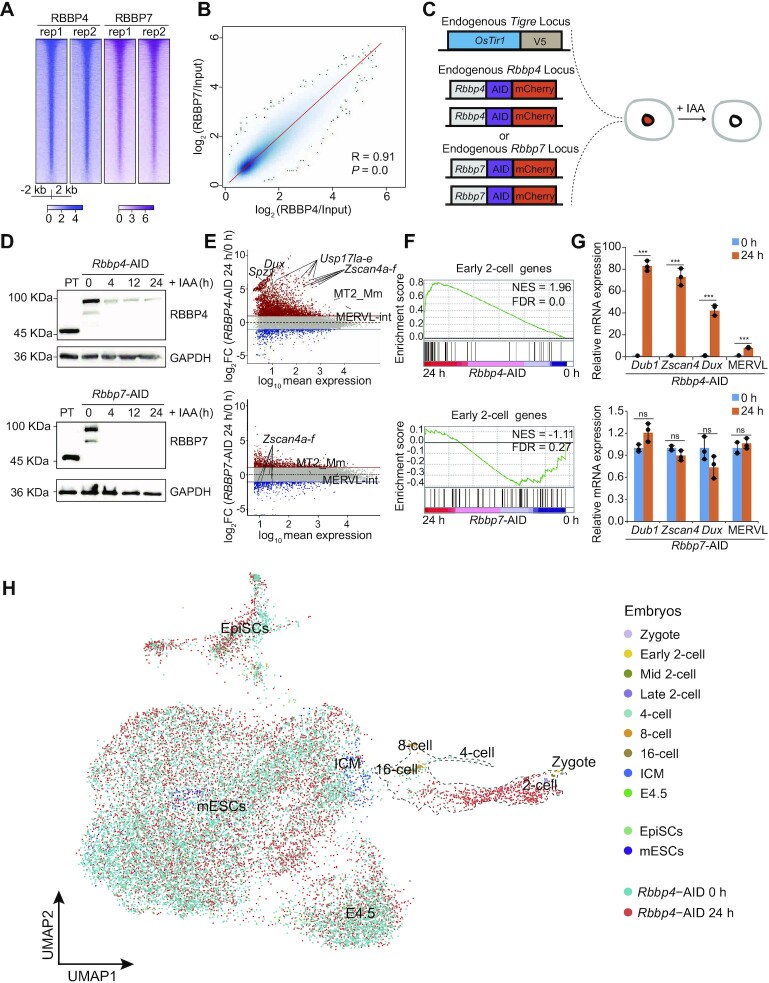
RBBP4, but not RBBP7, is indispensable for maintaining the identity of mESCs, and RBBP4 degradation activates 2C gene expression. (**A**) Heatmap of ChIP-seq signals for RBBP4 and RBBP7. (**B**) Pearson correlation of ChIP-seq signals between RBBP4 and RBBP7. (**C**) Schematic diagram of the AID system to degrade RBBP4 or RBBP7. The endogenous *Rbbp4* gene or *Rbbp7* gene was tagged with AID–mCherry in *OsTir1* knockin parental mESCs. (**D**) Western blots showing protein levels of RBBP4 and RBBP7 in cells treated with IAA at different time points. (**E**) MA plot showing the expression changes of genes and TEs after degradation of RBBP4 or RBBP7. The horizontal red and blue lines define log_2_FC >1 and < −1, respectively. (**F**) Gene set enrichment analysis (GSEA) of early-2C genes in RBBP4- or RBBP7-depleted RNA-seq data. (**G**) RT–qPCR to detect expression of 2C genes and MERVL elements following degradation of RBBP4 or RBBP7 (*n* = 3). (**H**) Comparison of our scRNA-seq data with previously published scRNA-seq data of mouse pre-implantation embryos from zygote to E4.5, naïve mESCs and EpiSCs.

To identify the similarities and differences in the chromatin binding and transcriptional regulation of RBBP4 and RBBP7, we attempted to perform ChIP-seq for RBBP4 and RBBP7 by using commercial antibodies. Unfortunately, these commercial anti-RBBP4 and anti-RBBP7 antibodies did not work properly for ChIP experiments. Therefore, we generated Biotin-tagged *Rbbp4* and *Rbbp*7 mESC lines (Supplementary Figure S1A), respectively, and performed Biotin-RBBP4 and Biotin-RBBP7 ChIP-seq experiments. Our data indicated that most of the binding sites for RBBP4 and RBBP7 were compatible (Figure 1A, B; Supplementary Figure S1B). To explore whether RBBP4 and RBBP7 are functionally redundant, we constructed an acute auxin-induced degron (AID) system to deplete either RBBP4 or RBBP7 protein in mESCs (Figure 1C). We integrated the AID–mCherry sequence at the C-terminus of the endogenous *Rbbp4* or *Rbbp7* gene locus by CRISPR/Cas9 genome editing (Supplementary Figure S1C, D) in a parental (PT) line encoding *OsTir1* (73); the resulting cell lines were referred to as *Rbbp4*-AID and *Rbbp7*-AID, respectively. The addition of IAA for 4 h successfully induced rapid depletion of the RBBP4 or RBBP7 protein (Figure 1D; Supplementary Figure S1E). RBBP4 depletion slowed the proliferation of mESCs and caused apoptosis (Supplementary Figure S1F, G). Moreover, RBBP4 depletion arrested cells at the G_1_ and S phases, demonstrating that RBBP4 is necessary for normal self-renewal of mESCs (Supplementary Figure S1H, I). Furthermore, RBBP4 depletion also resulted in a severe decrease in AP staining (Supplementary Figure S1J), leading to a differentiated phenotype and destruction of the normal mESC morphology (Supplementary Figure S1K). In contrast, RBBP7 depletion had little effect on cell proliferation, morphology and cell cycle of mESCs (Supplementary Figure S1F–K). Altogether, our data demonstrate that RBBP4 is necessary for maintaining the identity of mESCs, but that RBBP7 is dispensable.

### RBBP4, but not RBBP7, represses the transcriptional activity associated with the 2C-like cell program

To examine the effect of RBBP4 or RBBP7 on gene expression, we performed RNA-seq experiments after acute depletion of either RBBP4 or RBBP7, and PCA indicated high reproducibility between two biological replicates (Supplementary Figure S1L). A large number of genes were up-regulated after RBBP4 degradation, while only a few were altered after RBBP7 degradation (Supplementary Figure S1M). Moreover, the numbers and levels of differentially expressed genes increased with the duration of IAA treatment (Supplementary Figure S1M). Additionally, we observed that very few differentially expressed genes were shared between RBBP4-depleted and RBBP7-depleted cells (Supplementary Figure S1N). These results suggest that the degradation of RBBP4 and RBBP7 led to distinct gene expression patterns, which is consistent with the different phenotypes after depletion of RBBP4 and RBBP7 (Supplementary Figure S1F–K).

Intriguingly, we found that RBBP4 degradation significantly up-regulated the expression of *Zscan4* family members, *Dux* and the MERVL elements (including MT2_Mm and MERVL-int) (Figure 1E), which are typical 2C markers (27,28,74). Consistently, by comparing our RNA-seq data with previously published data on early-2C genes (75), we further observed that RBBP4 degradation strikingly activated the genes expressed at the 2C stage (Figure 1F; Supplementary Table S4). RT–qPCR confirmed the activation of 2C-specific genes (*Dub1*, *Zscan4* and *Dux*) and MERVL at 24 h after RBBP4 depletion (Figure 1G). Conversely, RBBP7 degradation had little effect on the expression of 2C-specific genes, as revealed by RNA-seq and RT–qPCR analyses (Figure 1E–G). To further demonstrate the role of RBBP4 in repressing the 2C gene program, MERVL-tdTomato reporter mESCs (76) were used to deplete *Rbbp4* and *Rbbp7* by using shRNA oligos (Supplementary Figure S2A). Our data indicated that the expression of MERVL and 2C-like genes (*Zscan4* and *Dub1*) and the population of MERVL-tdTomato cells were significantly increased in the *Rbbp4*-depleted cells (Supplementary Figure S2B–D), but not in *Rbbp7*-depleted cells (Supplementary Figure S2E–G).

To further examine whether RBBP4 degradation specifically promotes the transition of mESCs to 2CLCs, but not other embryonic stage-like cells, we performed scRNA-seq by using *Rbbp4*-AID mESCs cultured with or without IAA treatment, and integrated the data with published single-cell transcriptomes of mouse early embryos (77–80). UMAP showed that a fraction of cells (7.38%, 622/8432) (named totipotent-like cells) among the RBBP4-depleted cells were clustered closer to 2C embryos, representing an ∼7-fold increase over the corresponding fraction of cells without RBBP4 degradation (1.06%, 97/9122) (Figure 1H; Supplementary Figure S3A). The correlation between these totipotent-like cells and early embryos was recalculated and showed that these cells had a strong resemblance to mid- and late-2C embryos (Supplementary Figure S3B). Consistently, these totipotent-like cells expressed totipotency genes (such as *Zscan4*, *Usp17l*, *Gm13119* and *Gm4027*) at a high level and pluripotency genes (such as *Oct4* and *Nanog*) at a low level (Supplementary Figure S3C). Collectively, our results strongly support that RBBP4, but not RBBP7, plays a vital role in regulating the stem cell fate transition from pluripotency to totipotency.

### *Rbbp4* depletion enhances trophoblast formation

**Figure 2.**
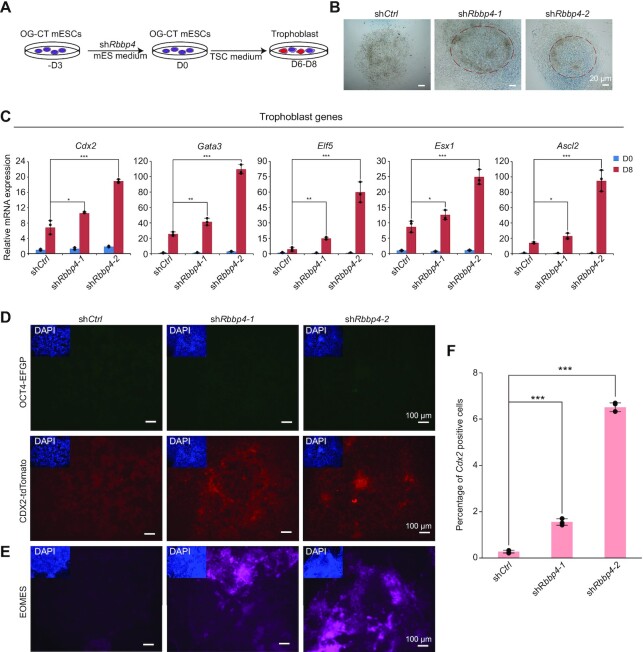
*Rbbp4* depletion enhances trophoblast formation. (**A**) Schematic diagram showing the conversion from OG-CT mESCs to trophoblasts. (**B**) Phase contrast on D8 of control and *Rbbp4*-depleted cells. Scale bar, 20 μm. (**C**) RT–qPCR to detect the expression levels of the trophoblast marker genes. (**D**) EGFP (OCT4) and tdTomato (CDX2) expression on D6 after inducing the transition from OG-CT mESCs to trophoblasts. Scale bar, 100 μm. (**E**) Immunostaining of EOMES on D6 after inducing the transition from OG-CT mESCs to trophoblasts. Scale bar, 100 μm. (**F**) Statistical analysis of the *Cdx2*-tdTomato positive ratios. Data shown in (C) and (F) represent the average of three independent experiments.

Since totipotent-like cells, but not mESCs, have the capacity to develop into extraembryonic lineages, we took advantage of *Oct4*-EGFP (enhanced green fluorescent protein) and *Cdx2*-tdTomato dual fluorescent reporter (OG-CT) mESCs (81) to explore whether trophoblast could be derived from the *Rbbp4*-depleted cells in mouse TSC medium (82) (Figure 2A). As expected, *Rbbp4-*depleted OG-CT mESCs activated the expression of 2C markers (MERVL and *Zscan4*) (Supplementary Figure S4A). mESCs cultured in TSC medium gradually formed epithelial-like cells. The differentiated cells derived by loss of *Rbbp4* were mostly multilayered and formed cellular aggregates, while the differentiated control mESCs exhibited a flat layered cellular structure (Figure 2B). We next analyzed the expression of pluripotency and trophoblast genes and observed that the expression of pluripotency genes (including *Oct4*, *Nanog*, *Sall4* and *Tdgf1*) was decreased in all differentiated cells (Supplementary Figure S4B) and the expression of trophoblast genes (including *Cdx2*, *Gata3*, *Elf5*, *Esx1* and *Ascl2* (22,24,83–85)) was dramatically increased only in *Rbbp4*-depleted cells but not in control cells (Figure 2C). Consistent with these findings, the expression of OCT4–EGFP was rarely observed in all differentiated cells, but the robust expression of CDX2–tdTomato and EOMES was observed only in *Rbbp4*-depleted cells (Figure 2D, E). Flow cytometry analysis showed that the proportion of *Cdx2*-positive cells produced by *Rbbp4-*depleted mESCs was increased by >4.5 times compared with that of the control cells (Figure 2F; Supplementary Figure S4C). Our results suggested that *Rbbp4* depletion enhanced cell fate conversion from mESCs to trophoblast lineages.

### RBBP4 regulates the expression of ERVs

**Figure 3.**
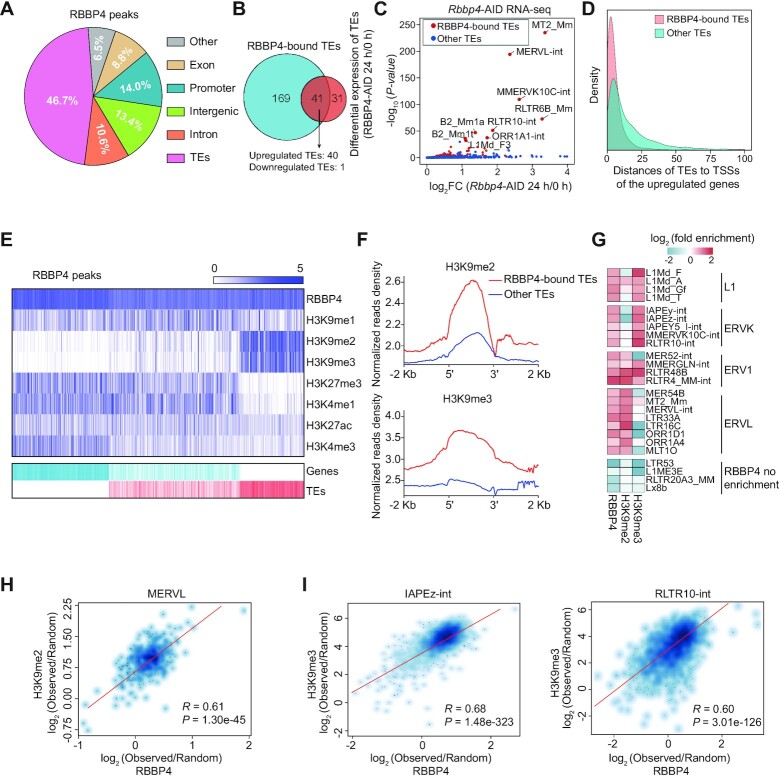
RBBP4 represses various TEs. (**A**) Genomic annotations of RBBP4 peaks. (**B**) Venn diagram showing the overlap between RBBP4-bound TEs and differentially expressed TEs after RBBP4 depletion. (**C**) Dot plot showing changes in TE expression following RBBP4 depletion. TEs bound by RBBP4 are labeled with red circles and other TEs are labeled with blue circles. TEs with significantly increased expression (*P* <1e-30) are highlighted. (**D**) Density plot showing the absolute distances from the TSSs of up-regulated genes after RBBP4 depletion to the nearest TEs bound by RBBP4. The distances to other TEs are used as background controls. (**E**) Heatmap showing the enrichment of RBBP4 and histone modifications on RBBP4-bound genes and TEs. (**F**) Average distribution of H3K9me2 and H3K9me3 signals at RBBP4-bound TEs versus other TEs. (**G**) Heatmap showing the enrichment of RBBP4, H3K9me2 and H3K9me3 at the indicated TEs. (**H**) Correlation analysis between RBBP4 and H3K9me2 on full-length MERVL elements (*n* = 535). (**I**) Correlation analysis between RBBP4 and H3K9me3 on IAPEz-int (stitched adjacent IAPEz-int fragments) (*n* = 2894) and RLTR10-int (*n* = 1587) elements. Statistical analyses in (H) and (I) are performed using the two-sided Pearson's correlation test.

To investigate how RBBP4 negatively regulates totipotency, we focused on the chromatin distribution of RBBP4 binding and observed that RBBP4 was mostly enriched at TEs (46.7%) and a small percentage at promoters (14%) (Figure 3A). Intriguingly, we found that RBBP4 depletion up-regulated 69 TE families (Supplementary Table S5), ∼58% (40/69) of which were directly bound by RBBP4 (Figure 3B); but only 7.5% (15/199) of up-regulated 2C genes were directly bound by RBBP4 (Supplementary Figure S5A). TEs, especially ERVs, are abundantly activated during zygote genome activation (ZGA), which is critical for embryonic totipotency, and can function as *cis*-regulatory elements to regulate expression of their neighboring genes (86,87). Interestingly, we found that RBBP4 peaks were mostly enriched at ERVs and long interspersed nuclear elements (LINEs) (Supplementary Figure S5B, C). Furthermore, among these RBBP4-bound TEs, ERVs, such as MERVL (ERVL), RLTR10-int (ERVK) and RLTR6B_Mm (ERV1), tended to exhibit much more increased expression after RBBP4 depletion (Supplementary Figure S5D, E). Notably, RBBP4-bound MERVL elements were most highly expressed after RBBP4 degradation (Figure 3C). Moreover, we observed that the transcription start sites (TSSs) of the up-regulated genes were in closer proximity to the RBBP4-bound TEs than to other TEs (Figure 3D). Additionally, the distances between the strongly up-regulated early-2C genes [log_2_FC >5, false discovery rate (FDR) <0.01] and RBBP4-bound TEs were <1 kb (Supplementary Figure S5F), suggesting that RBBP4 might control the expression of these 2C genes by regulating TEs.

To explore how RBBP4 regulates TEs, we conducted hierarchical clustering of RBBP4 peaks with both active (H3K4me1, H3K4me3 and H3K27ac) (88,89) and repressed (H3K9me1, H3K9me2, H3K9me3 and H3K27me3) (89–92) histone marks over genes and TE regions and observed that RBBP4 was mostly associated with two repressive histone marks H3K9me2 and H3K9me3 at TE sites, respectively (Figure 3E; Supplementary Figure S5G, H). Remarkably, strong enrichment of H3K9me2 and H3K9me3 at RBBP4-bound TEs relative to other TE sites was observed (Figure 3F). The enrichment analyses showed that RBBP4 and H3K9me2 were mainly enriched at ERVL sites, and RBBP4 and H3K9me3 were mainly enriched on ERVK, ERV1 and LINE sites (Figure 3G). Specifically, RBBP4 binding densities were positively correlated with H3K9me2 on MERVL (ERVL) elements (Figure 3H), and with H3K9me3 on IAPEz-int and RLTR10-int (ERVK) elements (Figure 3I). Together, these data demonstrate that RBBP4 globally represses the transcription of ERVs, and co-binds together with heterochromatin marks either H3K9me2 at ERVL sites or H3K9me3 at ERVK sites.

### RBBP4 is required for heterochromatin formation

**Figure 4.**
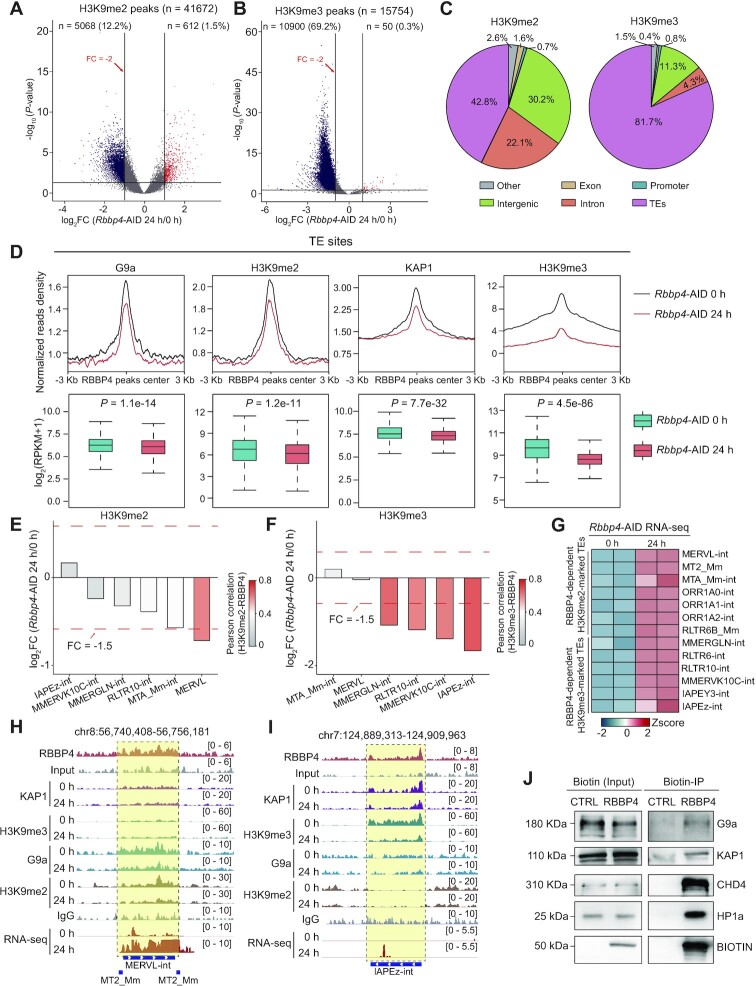
RBBP4 is required for heterochromatin formation. (A and B) Volcano plots showing the binding changes of H3K9me2 (**A**) and H3K9me3 (**B**) following RBBP4 depletion. (**C**) Genomic annotations of decreased binding sites of H3K9me2 (left) and H3K9me3 (right) after RBBP4 degradation. (**D**) Pile ups and boxplots showing H3K9me2, H3K9me3, G9a and KAP1 signals at the RBBP4-bound TE sites in mESCs with or without RBBP4 depletion. (E and F) Bar plots showing the fold changes of H3K9me2 (**E**) and H3K9me3 (**F**) enrichment on different ERVs upon RBBP4 depletion. The color bar illustrates the Pearson correlation between RBBP4 and H3K9me2 (E) or between RBBP4 and H3K9me3 (F). (**G**) Heatmap showing the expression changes of the indicated TEs after RBBP4 depletion. (**H** and **I**) Selected genomic views of RBBP4, H3K9me2, H3K9me3, G9a and KAP1 signals for the indicated TEs in mESCs with or without RBBP4 depletion. (**J**) Biotin-immunoprecipitation assays showing the interaction of RBBP4 with G9a, KAP1 and CHD4.

To investigate whether heterochromatin formation is dependent of RBBP4, we identified the binding sites for H3K9me2 and H3K9me3 in *Rbbp4*-AID mESCs with or without IAA treatment. Our data indicated that RBBP4 degradation resulted in the significant reduction of 5068 (12%) H3K9me2 peaks (42.8% in TEs sites) and 10900 (69%) H3K9me3 peaks (81.7% in TEs sites), but caused an increase of only 612 (1.5%) H3K9me2 peaks and 50 (0.3%) H3K9me3 peaks, respectively (Figure 4A–C). Moreover, pile ups and boxplots revealed that RBBP4 loss significantly reduced binding of H3K9me2 and H3K9me3 on RBBP4-bound TE sites (Figure 4D; Supplementary Tables S6 and S7). Specifically, we further observed that RBBP4 degradation attenuated H3K9me2 enrichment on RBBP4–H3K9me2-co-bound ERVL, particularly MERVL (Figure 4E), and H3K9me3 enrichment on RBBP4–H3K9me3-co-bound ERV1/ERVK (such as MMERGLN-int and IAPEz-int) (Figure 4F). Consistently, the expression of RBBP4-dependent H3K9me2-marked and H3K9me3-marked TEs after RBBP4 depletion was significantly enhanced (Figure 4G).

At least five H3K9-specific methyltransferases have been described in mammals: SUV39H1, SUV39H2, SETDB1, G9a and GLP (93). Next, we sought to determine whether RBBP4 establishes H3K9me2/3 on these TEs by regulating H3K9 methyltransferases or the associated factors. Using the ProteomicsDB database (94), we found that the protein expression level of RBBP4 showed a strong correlation with that of KAP1 (*R* = 0.80, *P* = 2.95e-22), which recruits SETDB1 to the target sites (95), with that of G9a (*R* = 0.70, *P* = 6.29e-15) and with that of GLP (*R* = 0.70, *P* = 9.38e-15), but was relatively weakly correlated with the expression levels of other lysine methyltransferases (*R* < 0.6) (Supplementary Figure S6A). Moreover, our data indicated that KAP1-binding sites were highly correlated with RBBP4-dependent H3K9me3-marked TEs and that G9a-binding sites were highly associated with RBBP4-dependent H3K9me2-marked TEs (Supplementary Figure S6B). Furthermore, PCA revealed that RBBP4 depletion in mESCs led to similar gene expression patterns to both *Kap1* knockout (96) and *G9a* knockout (97), which resulted in a gene expression signature similar to that of previously reported 2CLCs (29,98) (Supplementary Figure S6C). These results suggest that both KAP1 and G9a might be crucial for RBBP4-mediated heterochromatin formation.

More importantly, RBBP4 depletion had no significant influence on the protein levels of G9a and KAP1 (Supplementary Figure S6D), but diminished their binding to RBBP4-bound sites (Figure 4D). Furthermore, RBBP4 degradation resulted in a reduction of binding not only of G9a at ERVL elements but also of KAP1 at ERVK elements (Supplementary Figure S6E–G). Specifically, the occupancy of both G9a on MERVL sites and KAP1 on IAPEz-int sites was significantly diminished (Figure 4H, I; Supplementary Figure S6H). These data suggest that RBBP4 is an upstream regulator of both KAP1 and G9a in regulating TEs. Additionally, Biotin-immunoprecipitation analysis showed that RBBP4 interacted with both G9a and KAP1 (Figure 4J). Together, these results indicate that RBBP4 could regulate heterochromatin establishment in mESCs by recruiting KAP1 and G9a.

### AID-mediated RBBP4 degradation reduces nucleosome occupancy at the regions of ERVK and ERVL within heterochromatin

**Figure 5.**
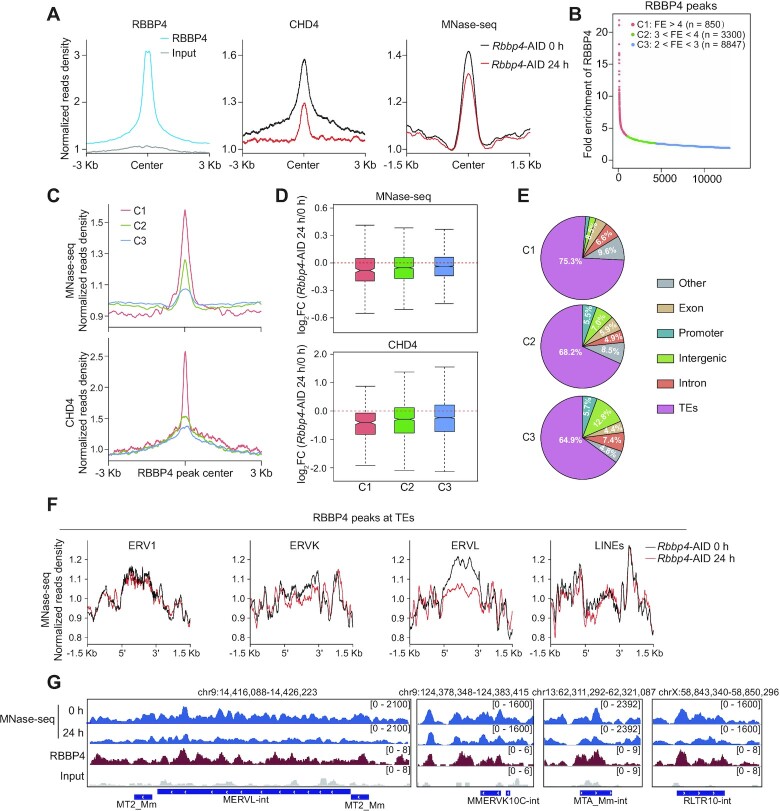
RBBP4 loss reduces nucleosome occupancy at heterochromatin regions. (**A**) Changes in CHD4 binding and nucleosome occupancy (MNase-seq) at RBBP4 peaks after RBBP4 depletion. (**B**) Scatter plot showing the fold enrichment (FE) of RBBP4 peaks. (**C**) Read density pile ups of nucleosome occupancy and CHD4 at different fold-enriched RBBP4-bound sites. (**D**) Changes in the nucleosome occupancy and CHD4 binding at different fold-enriched RBBP4-bound sites. The box plots denote medians and interquartile ranges (IQRs). (**E**) Distribution of reduced nucleosome regions across different fold-enriched RBBP4-bound sites after RBBP4 depletion. (**F**) Read density pile ups showing the intensity of MNase-seq signals at nucleosome-occupied regions of RBBP4-bound TEs with or without RBBP4 depletion. (**G**) Selected genomic views of nucleosome occupancy at the indicated TEs.

RBBP4 is also a component of the NuRD complex (39), and CHD4 within this complex is responsible for remodeling chromatin structure in an ATP-dependent manner (99,100). Indeed, our Biotin–RBBP4 co-immunoprecipitation experiments indicated that RBBP4 also interacted with CHD4 (Figure 4J). Next, we performed MNase-seq to determine the effects of RBBP4 on nucleosome organization and noticed that RBBP4 degradation attenuated nucleosome occupancy around RBBP4-binding sites and decreased CHD4 binding (Figure 5A; Supplementary Figure S7A). Moreover, we found that 2C genes and 2C-associated TEs (including MERVL) were up-regulated in *Chd4*-depleted mESCs by analyzing previously published RNA-seq data (101) (Supplementary Figure S7B, C). To understand the relationship between RBBP4 and local nucleosome density in more depth, we split the RBBP4 peaks into three classes (C1–C3) according to the RBBP4 enrichment strength (Figure 5B). Our data showed that the strongest RBBP4-bound C1 sites had the highest intensity of nucleosome occupancy and CHD4 binding (Figure 5C). Correspondingly, the reduction of nucleosome density and CHD4 binding was positively correlated with the RBBP4 binding strength (Figure 5D). Moreover, ∼75.3, 68.2 and 64.9% of the decreased nucleosome occupancy at C1, C2 and C3 sites, respectively, was located at TE regions (Figure 5E). In addition, our data showed that nucleosome occupancy was reduced at the RBBP4-bound ERVK (RLTR10-int and MMERVK10C-int) and ERVL (MERVL and MTA_Mm-int) sites, whereas nucleosome occupancy at RBBP4-bound ERV1 and LINE sites was unaffected (Figure 5F, G; Supplementary Figure S7D). Together, these results demonstrate that RBBP4 could recruit CHD4 to remodel nucleosome occupancy at the ERVK and ERVL sites within heterochromatin regions.

## DISCUSSION

Understanding how totipotent cells are generated will provide more insight for stem cell biology and promote the progress of regenerative medicine. Here, we show that the histone chaperone RBBP4 acts as a powerful barrier by regulating heterochromatin assembly for reprogramming of the pluripotent state toward the totipotent 2C-like state. RBBP4 recruits different epigenetic factors to distinct TEs and modulates chromatin compaction through chromatin remodelers.

Approximately 40% of the mouse genome is composed of TEs, of which approximately a quarter are ERVs (95). ERVs are grouped into three classes based on sequence similarity with different exogenous retroviruses (93), and their regulatory mechanisms are quite complicated. Distinct classes of ERVs are regulated by different chromatin marks and associated epigenetic factors. For instance, KAP1 has been identified as a key repressor of ERVK elements in mESCs and early embryonic development (95,102). KAP1 functions as a scaffold for multiple repressive partners, such as the KRAB-ZFP proteins, SETDB1 and METTL3 (96,103,104). In contrast, silencing of ERVL elements by chromatin-associated factors has not been systematically characterized. Although G9a and H3K9me2 have been shown to repress MERVL elements (105,106), how they are recruited to genomic targets and form a regulatory network is not fully understood.

Histone chaperones are responsible for histone deposition onto DNA to form chromatin during various processes, such as DNA replication, transcription and repair (107,108). Intriguingly, we found that the histone chaperone RBBP4 binds to ERVs and functions as an upstream factor to recruit G9a and KAP1, followed by deposition of H3K9me2 and H3K9me3 at ERVL and ERVK elements, respectively. Furthermore, RBBP4 interacts with HP1 (Figure 4J), which is important for heterochromatin spreading (109). Therefore, we conclude that RBBP4 acts as a critical regulator for heterochromatin establishment and possible spreading.

In addition to RBBP4, the two largest subunits (CHAF1A and CHAF1B) of the CAF-1 complex have also been shown to regulate the establishment of heterochromatin (110–112). CHAF1A/B could inhibit the expression of unintegrated HIV-1 DNA (112), but RBBP4 only represses the transcription of integrated HIV-1 proviral DNA (113). Therefore, the biological functions of RBBP4 and CHAF1A/B in regulating heterochromatin formation might be independent or complementary. The relationships between RBBP4 and the CAF-1 complex still need further exploration.

ATP-dependent chromatin remodelers (including four families CHD, SWI/SNF, ISWI and INO80) can slide, eject, insert or replace histones within nucleosomes to alter chromatin structure and accessibility (70). In the totipotent cells, chromatin is highly relaxed, especially at TE regions (75,114), but it still remains unclear what chromatin remodelers do. Our results show that RBBP4 depletion leads to the changes in colony morphologies of mESCs, which is consistent with *Chd4* deficiency (115). Moreover, RBBP4 depletion activates TEs, such as MERVL, which is similar to loss of CHD4 (101). Importantly, RBBP4 depletion reduces CHD4 enrichment and nucleosome occupancy around TEs (Figure 5). RBBP4 and CHD4 co-exist in the NuRD complex, which also contains the deacetylase HDAC1/2 and other accessory proteins (116,117). Surprisingly, we observed that the total H3 acetylation level was not changed after acute degradation of RBBP4 (data not shown), suggesting that histone acetylation is dispensable for transcriptional activation in mESCs. In addition, CHD4 is also a member of the ChAHP complex (comprising CHD4, ADNP and HP1), which plays an important role in heterochromatin organization (118,119). This direct and indirect evidence suggests that RBBP4 plays vital roles in repressive chromatin deposition and remodeling.

A recent study showed that mouse totipotent cells and 2CLCs exhibit much slower DNA replication speed than pluripotent stem cells (120). Our results showed that the cell proliferation rate is retarded in RBBP4-depleted mESCs and the cell cycle is arrested at the G_1_ and S phases along with a global decrease in the G_2_ phase (Supplementary Figure S1I). These results also suggest that RBBP4-depleted mESCs acquire features similar to 2CLCs. RBBP4 depletion changes the cell cycle, possibly because RBBP4 also exists in DNA replication-modulating complexes, including DREAM, CAF-1 and CRL4 (121–123), and RBBP4 has been found at the replication forks (124). In addition, the NuRD chromatin remodeling complex might be involved in heterochromatin assembly during S phase, and loss of CHD4 results in a slow-growth phenotype with delayed S phase progression (125,126). These results indicate that RBBP4 and CHD4 might work in concert in heterochromatin assembly and epigenetic inheritance through DNA replication.

Our results show that RBBP4, but not its homolog RBBP7, is required for maintaining the identity of mESCs, which is consistent with a recent study that used a different RBBP4/RBBP7 deletion strategy (46). We further found that RBBP4 but not RBBP7 is a repressor of 2C genes, which coincides with the different temporal and spatial expression patterns of RBBP4 and RBBP7 during pre-implantation embryo development. The expression level of RBBP4 is lower at the mouse early 2C stage than at the other pre-implantation stages, but RBBP7 is highly expressed in oocytes and gradually decreased in later stages (data not shown). Our data suggest that RBBP4 and RBBP7 are not redundant in mESCs. The expression levels of RBBP4 and RBBP7 also vary between different tissues in mice (40), and RBBP4 and RBBP7 only have one ortholog: p55 in *Drosophila* and RebL1 in *Tetrahymena thermophila*, respectively (127,128). The evolutionary variation among different species and the different expression patterns of RBBP4 and RBBP7 might explain such functional diversity in higher eukaryotes.

**Figure 6.**
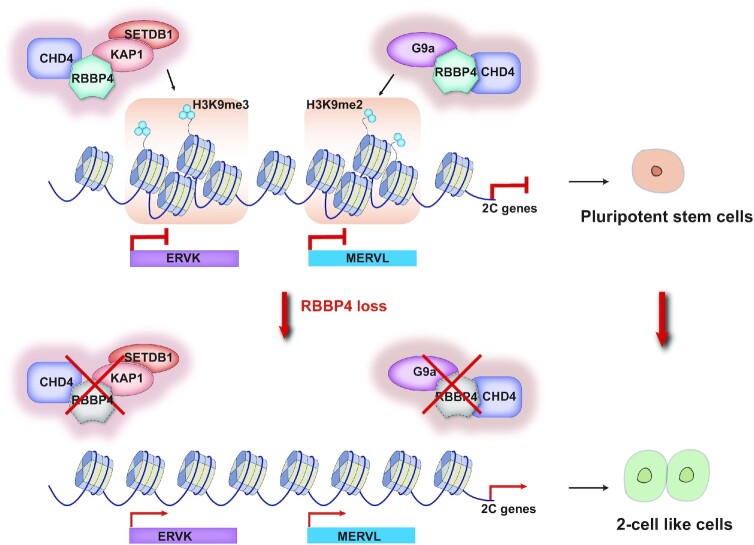
Model of RBBP4-dependent heterochromatin assembly and pluripotency to totipotency transition upon RBBP4 loss. RBBP4 binds to ERVs and functions as an upstream factor to recruit G9a and KAP1 and then deposit H3K9me2 and H3K9me3, respectively. Moreover, RBBP4 facilitates nucleosome occupancy through recruiting the chromatin remodeler CHD4 at heterochromatin regions. Therefore, RBBP4 depletion leads to the relaxation of repressive chromatin structure and activates expression of TEs (ERVs) and 2C genes to reprogram the pluripotent stem cells toward totipotent 2CLCs.

Overall, this study illustrates that RBBP4 plays a vital role in regulating heterochromatin assembly in mESCs and that loss of RBBP4 activates expression of a group of TEs and 2C genes to reprogram stem cell fate from pluripotency to totipotency (Figure 6).

## DATA AVAILABILITY

The RNA-seq, ChIP-seq, CUT&RUN, CUT&Tag and MNase-seq data in this study were deposited at the NCBI GEO under accession no. GSE218656 and the CNCB Genome Sequence Archive (GSA) under accession no. CRA007021. The previously published RNA-seq sequencing data that were reanalyzed here are available in the GEO under the accession codes: GSE49669 (97), GSE124580 (129), GSE75751 (98), GSE85627 (29) and GSE74278 (96). The previously published ChIP-seq data used in this paper were obtained from GSE153651 (92), GSE54412 (90), GSE149080 (91), GSE42152 (89) and GSE142519 (88). Smart-seq2 datasets were downloaded from GSE84892 (77), GSE100597 (80), GSE45719 (79) and GSE74155 (78). All other resources can be made available by the corresponding authors upon request.

## Supplementary Material

gkad219_Supplemental_Files
