# Supplementary material for: RBBP4 is an epigenetic barrier for the induced transition of pluripotent stem cells into totipotent 2C-like cells
Source: Nucleic Acids Res. 2023 Apr 6;51(11):5414–31. doi: 10.1093/nar/gkad219 (PMC10287929; doi:10.1093/nar/gkad219)
Supplement: gkad219_Supplemental_Files [file gkad219_supplemental_files.zip › Supplementary figure legends-revision.docx]

**SUPPLEMENTARY FIGURE LEGENDS**

**Supplementary Figure S1.** RBBP4, but not RBBP7, is necessary for maintaining the identity of mESCs. (**A**) Western blot showing protein levels of Biotin, RBBP4 and RBBP7 in wild-type mESCs and mESCs with either Biotin-tagged RBBP4 or Biotin-tagged RBBP7. GAPDH is used as a loading control. (**B**) Selected genomic views of RBBP4 and RBBP7 binding sites from ChIP-seq. (**C**) Homologous recombination strategy for tagging the endogenous *Rbbp4* or *Rbbp7* gene with AID-mCherry and a NeoR/KanR resistance gene. (**D**) PCR-based genotyping assay to validate generation of the *Rbbp4*-AID and *Rbbp7*-AID cell lines. (**E**) FACS analysis of mCherry fluorescence intensity in *Rbbp4*- or *Rbbp7*- AID-mCherry mESCs following different IAA treatment times. (**F**) Cell proliferation analysis of *Rbbp4*-AID*,* *Rbbp7*-AID and parental (PT) cell lines with or without IAA treatment. Data are presented as the mean ± s.d. (n = 6 independent wells). (**G**) Annexin V to analyze cellular apoptosis of *Rbbp4*-AID*,* *Rbbp7*-AID and PT cell lines with or without IAA treatment for 24 h. (**H**) Cell cycle analysis of *Rbbp4*-AID*,* *Rbbp7*-AID and PT cell lines with or without IAA treatment. (**I**) Histograms showing quantification of cycle phases based on DAPI analysis. (**J**) Alkaline phosphatase (AP) staining of untreated and 3-day IAA-treated *Rbbp4*-AID*,* *Rbbp7*-AID and PT cell lines. (**K**) Brightfield images of mESC colonies after IAA treatment at different time points. Scale bar, 100 μm. (**L**) PCA of RNA-seq data for mESCs after depleting RBBP4 or RBBP7 at different time points. (**M**) Number of differentially expressed genes in mESCs after depletion of RBBP4 or RBBP7 at different time points. (**N**) Venn diagram showing the overlap among the altered genes at 24 h after depleting RBBP4 or RBBP7. Data shown in (**G)** and (**I**) represent the average of three independent experiments.

**Supplementary Figure S2.** Depletion of *Rbbp4* but not *Rbbp7*, promotes the transition from mESCs to 2CLCs. (**A**) Schematic diagram of depleting *Rbbp4* or *Rbbp7* in mESCs containing the MERVL-tdTomato reporter. (**B** and **E**) RT–qPCR to detect the expression of MERVL and 2C genes (*Zscan4* and *Dub1*) in control and *Rbbp4*-depleted (**B**) as well as *Rbbp7*-depleted (**E**) MERVL-tdTomato reporter mESCs. **(C and F)** Flow cytometry analysis showing the percentage of MERVL-tdTomato-positive cells after *Rbbp4* knockdown (**C**) and *Rbbp7* knockdown (**F**). (**D** **and G**) Statistical analysis of the positive ratios in control and *Rbbp4*-depleted (**D**) as well as *Rbbp7*-depleted (**G**) MERVL-tdTomato reporter mESCs. Data shown in (**B**), (**D**), (**E**) and (**G**) represent the average of three independent experiments.

**Supplementary Figure S3.** *Rbbp4* depletion promotes the transition from mESCs to totipotent -like cells. (**A**) Cell ratios in different clusters of *Rbbp4*-AID mESCs with or without IAA treatment. (**B**) Heatmap showing the correlation between the data from totipotent-like cells and the published early embryo Smart-seq2 datasets. (**C**) Gene expression analysis showing the expression of totipotency and pluripotency genes in different cell types.

**Supplementary Figure S4.** *Rbbp4* depletion promotes the transition from mESCs to trophoblast. (**A**) RT–qPCR to detect the expression of *Rbbp4*, MERVL and *Zscan4* after *Rbbp4* depletion in OG-CT mESCs. (**B**) RT–qPCR showing the expression levels of the pluripotency genes on D0 and D8, respectively. (**C**) Flow cytometry analysis showing the percentage of *Cdx2*-tdTomato positive cells with or without *Rbbp4* depletion. Data shown in (**A**) and (**B**) represent the average of three independent experiments.

**Supplementary Figure S5.** RBBP4 binds to various TEs. (**A**) Venn diagram showing the overlap among the three gene clusters (RBBP4-bound genes, upregulated genes after RBBP4 degradation and 2C-specific genes). (**B**) Bar graph showing the overlap ratios of RBBP4 peaks with the transposable elements. (**C**) Box plots showing RBBP4 enrichment at selected TE elements. (**D**) RNA-seq analysis showing changes in expression of RBBP4-bound TEs after RBBP4 depletion. (**E**) Box plots showing changes in expression of RBBP4-bound indicated TEs following RBBP4 deletion. (**F**) Bar graph showing changes in expression of early 2C genes close to RBBP4-bound TEs within 2 kb. (**G**) Average distribution of RBBP4 and the selected histone modification signals at RBBP4-bound genes/TEs versus other genes/TEs. (**H**) Selected genomic views showing co-enrichment of RBBP4 with H3K9me2 and H3K9me3 at different TEs, respectively.

**Supplementary Figure S6.** RBBP4 regulates heterochromatin formation. (**A**) Pearson correlation coefficient for the protein levels of H3K9me2 and H3K9me3 modifiers with RBBP4 in the ProteomicsDB database. GAPDH is used as a negative control. The dotted line denotes *R* = 0.6. (**B**) Bar graph showing the Jaccard coefficient of binding peaks for H3K9me2 and H3K9me3 modifiers with RBBP4-dependent H3K9me2-marked TEs and with RBBP4-dependent H3K9me3-marked TEs. (**C**) PCA of RNA-seq data for RBBP4-depleted, *G9a*-KO, *Kap1*-KO, *Ezh2*-KO mESCs and other 2CLCs described in previous studies. (**D**) Western blot showing protein levels of RBBP4, G9a, KAP1, and CHD4 in *Rbbp4*-AID mESCs without or with IAA treatment for 24 h. (**E**) Aggregation plot showing changes in average enrichment of G9a or KAP1 at RBBP4-bound TE sites. (**F** and **G**) Box plots showing enrichment changes of G9a and KAP1 at ERVL and ERV1/ERVK elements, respectively, after RBBP4 depletion. (**H**) Aggregation plot showing changes in average enrichment of G9a at MERVL elements and KAP1 at IAPEz-int elements.

**Supplementary Figure S7.** RBBP4 facilitates nucleosome occupancy at TEs. (**A**) Dot plot showing change of CHD4 binding following RBBP4 depletion. (**B**) Venn diagram showing the overlap among upregulated genes after RBBP4 depletion and upregulated genes after *Chd4* KO and 2C-specific genes. (**C**) Heatmap showing expression changes of the indicated TEs and 2C genes after RBBP4 depletion and *Chd4*-KO. (**D**) Reads density pileups showing the intensity of MNase-seq signals at nucleosome-occupied regions of the indicated TEs with or without RBBP4 depletion.
