# Supplementary material for: RBBP4 is an epigenetic barrier for the induced transition of pluripotent stem cells into totipotent 2C-like cells
Source: Nucleic Acids Res. 2023 Apr 6;51(11):5414–31. doi: 10.1093/nar/gkad219 (PMC10287929; doi:10.1093/nar/gkad219)

Supplementary Figure S1

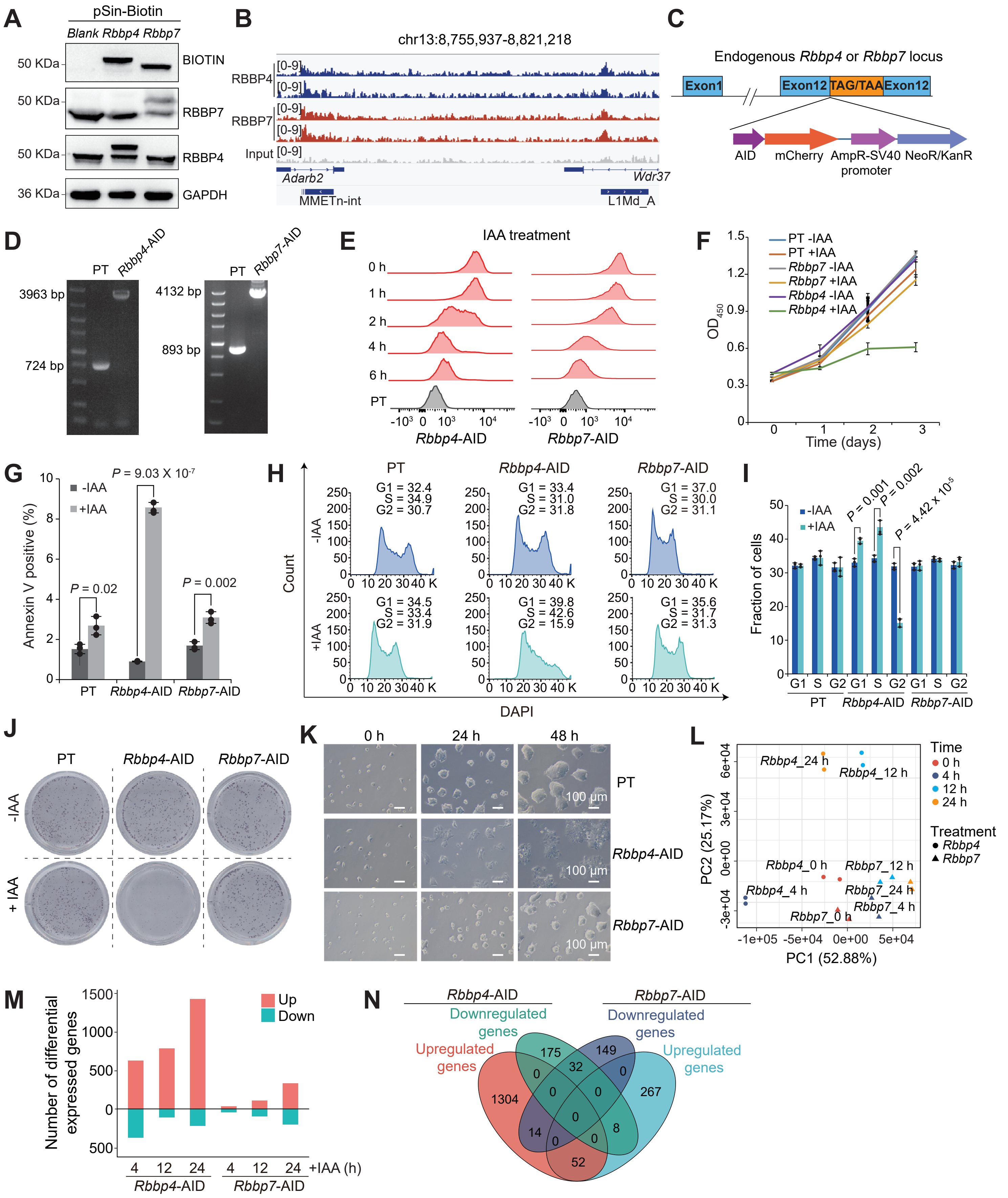

Supplementary Figure S2

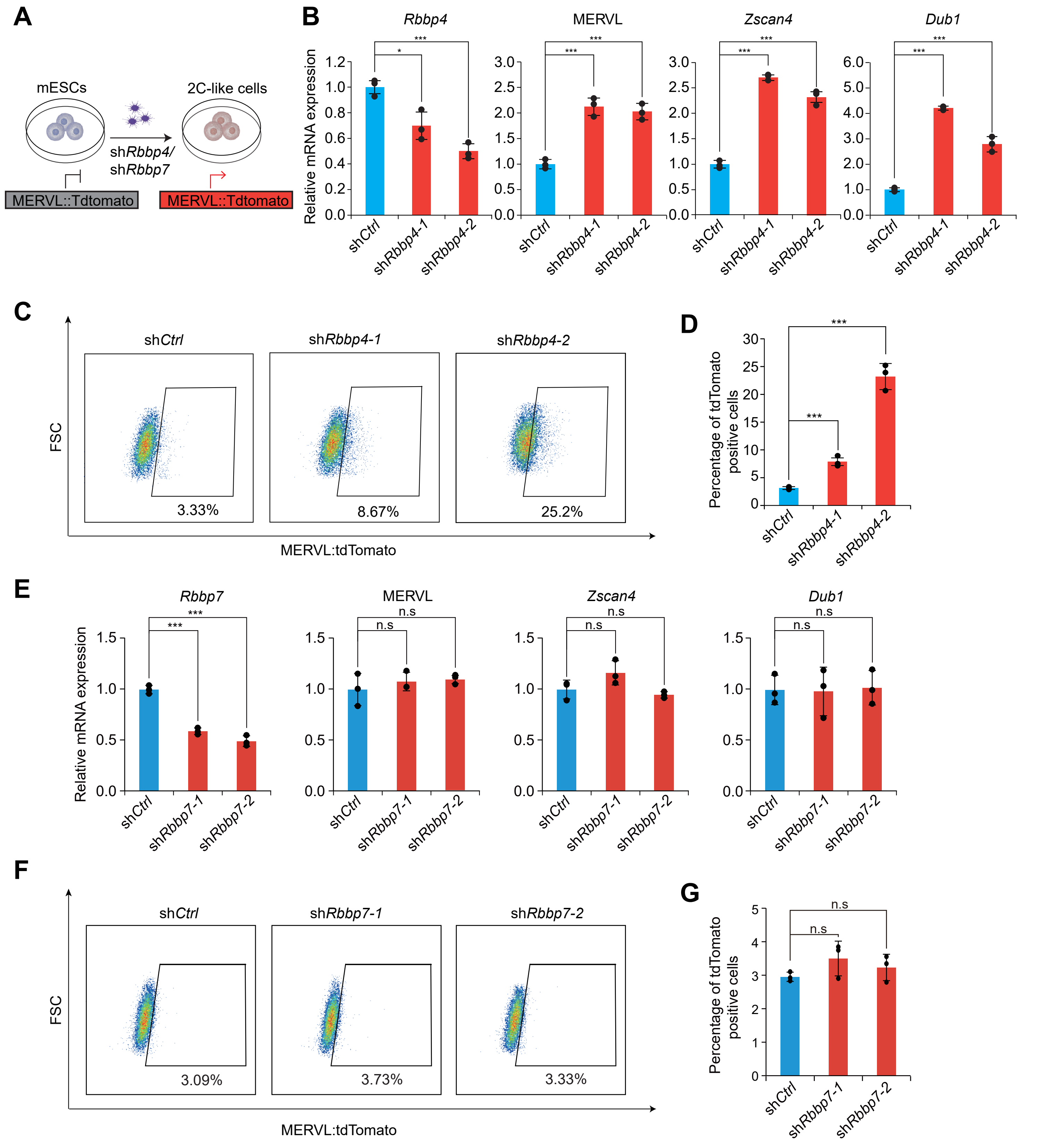

Supplementary Figure S3

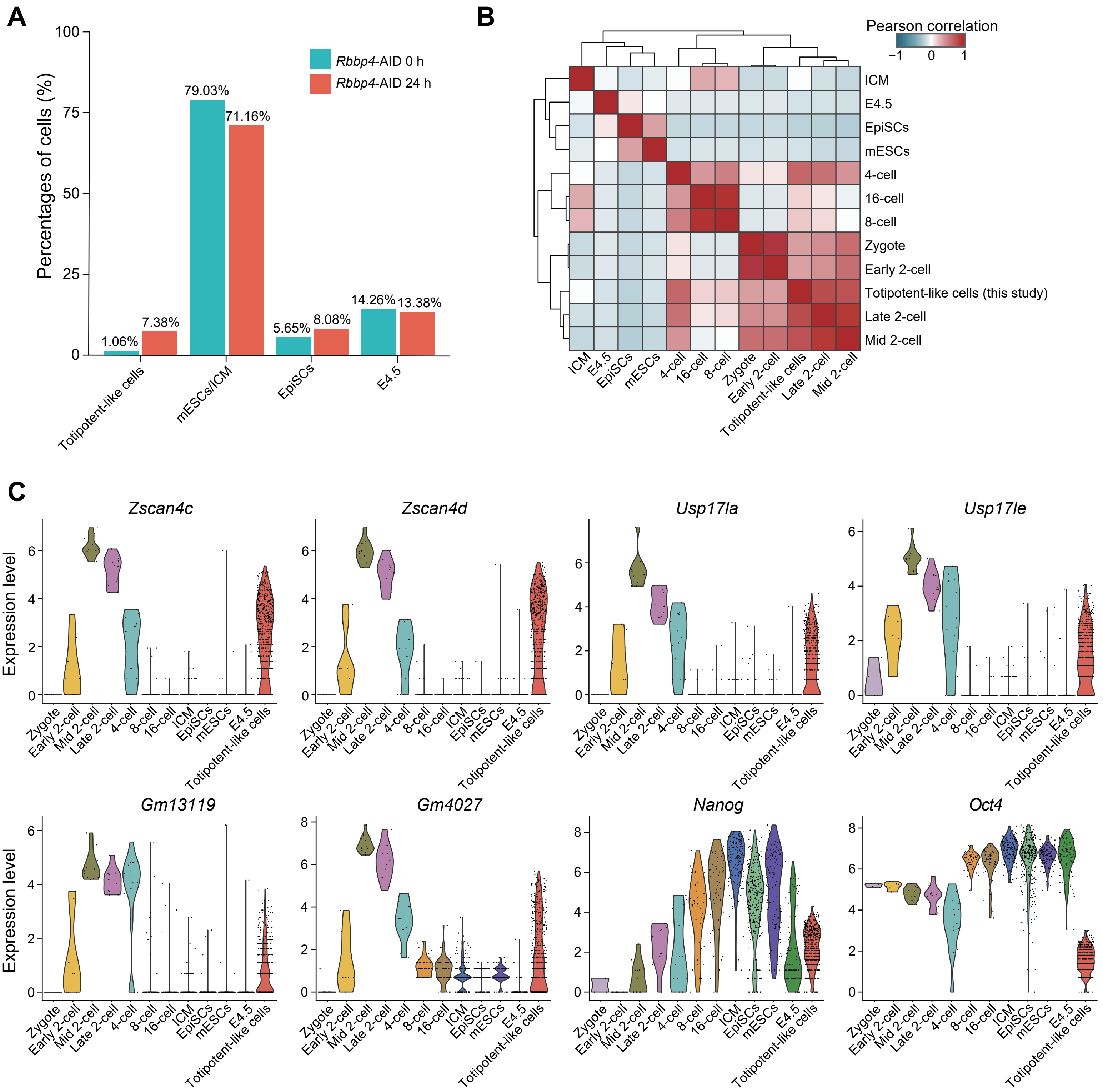

Supplementary Figure S4

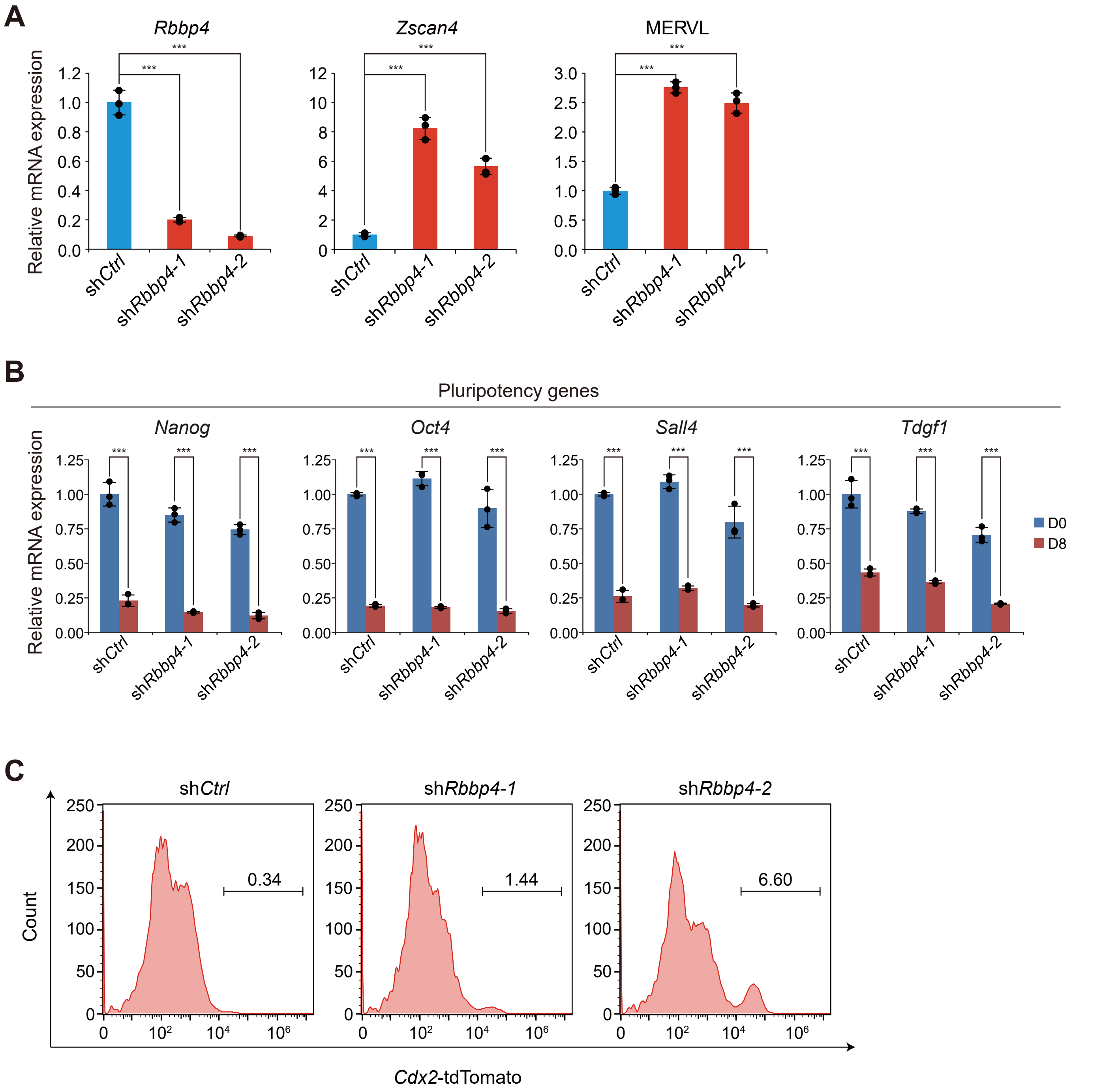

Supplementary Figure S5

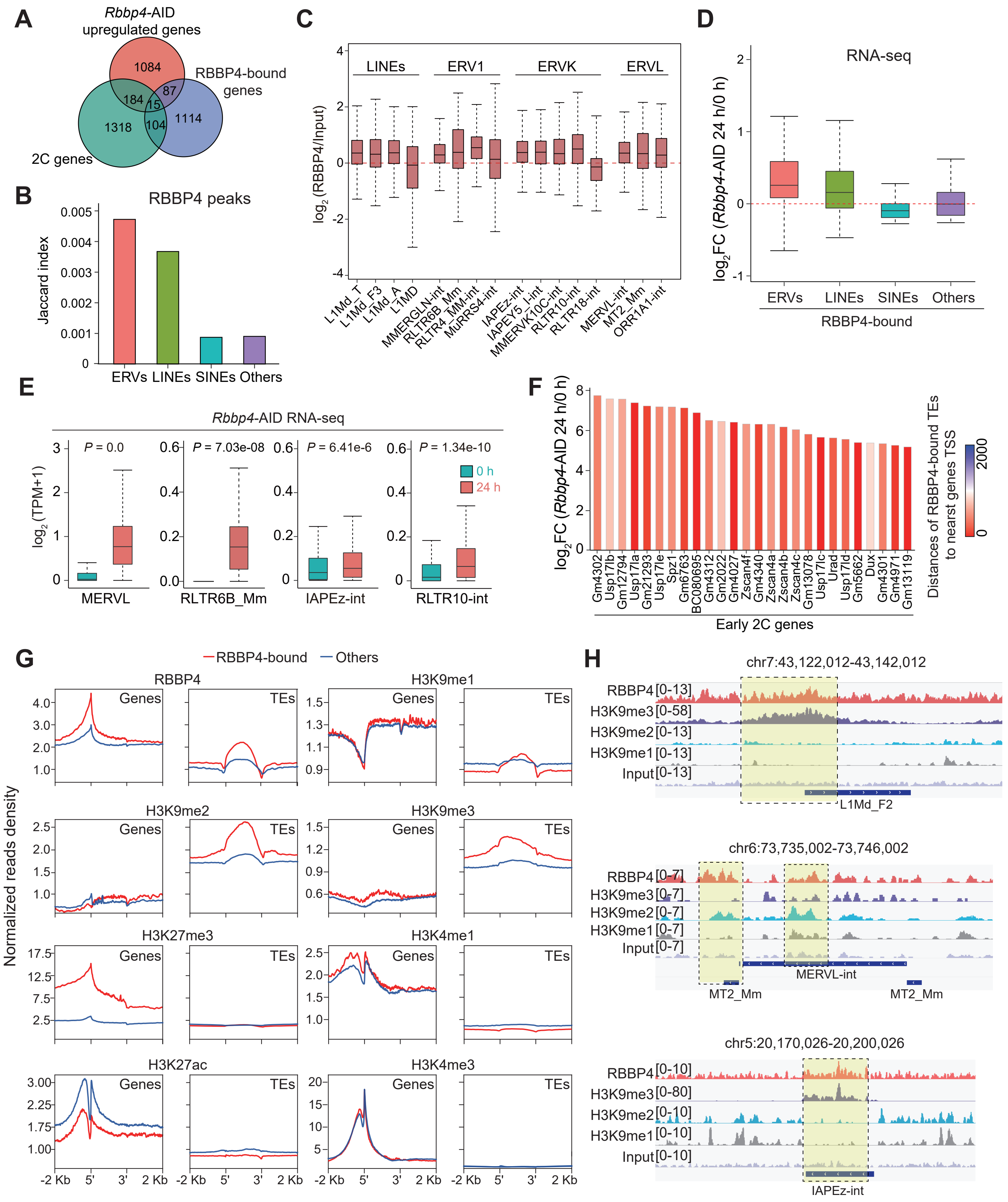

Supplementary Figure S6

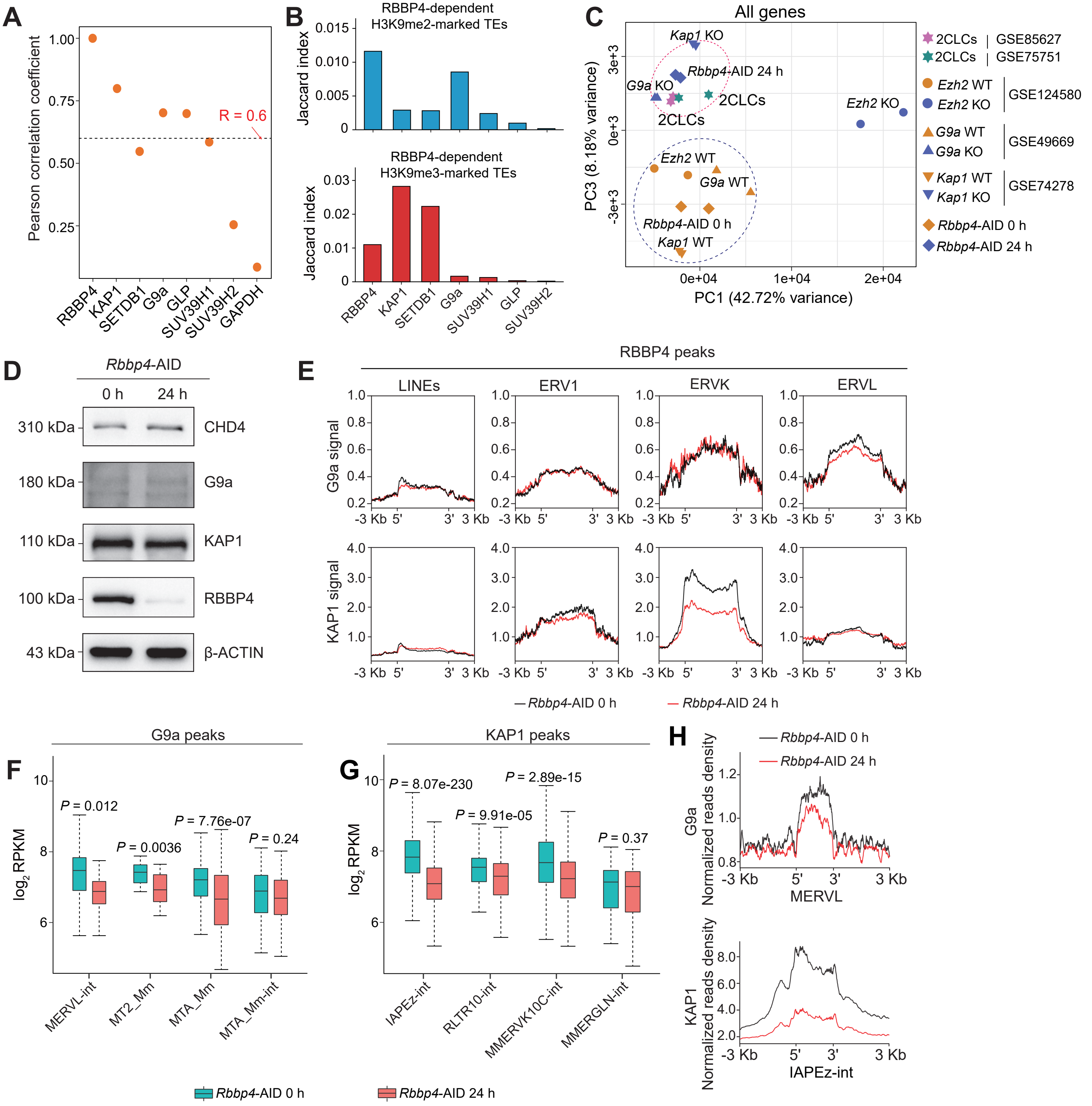

Supplementary Figure S7

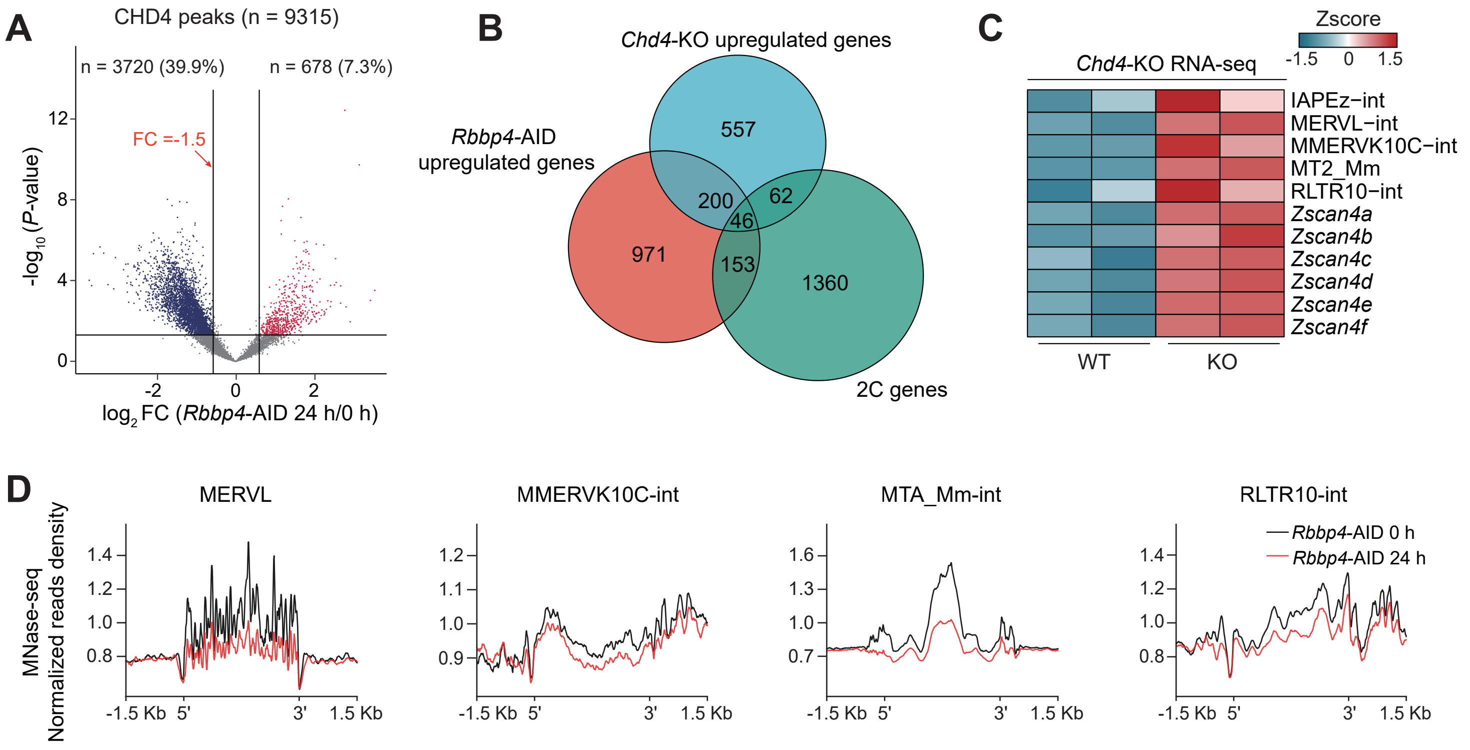

Supplement: gkad219_Supplemental_Files [file gkad219_supplemental_files.zip › Supplementary Figures.pdf]
